# Polysomal mRNA Association and Gene Expression in *Trypanosoma brucei*

**DOI:** 10.12688/wellcomeopenres.16430.1

**Published:** 2021-02-22

**Authors:** Michele Tinti, Anna Kelner-Mirôn, Lizzie J. Marriott, Michael A.J. Ferguson

**Affiliations:** 1 Wellcome Centre for Anti-Infectives Research (WCAIR), School of Life Sciences, University of Dundee, Dundee, Dundee, UK

**Keywords:** RNA-seq, mRNA, Polysome, Trypanosoma brucei, Bloodstream form, Procyclic form, machine learning

## Abstract

**Background**: The contrasting physiological environments of *Trypanosoma brucei* procyclic (insect vector) and bloodstream (mammalian host) forms necessitates deployment of different molecular processes and, therefore, changes in protein expression. Transcriptional regulation is unusual in *T. brucei* because the arrangement of genes is polycistronic; however, genes which are transcribed together are subsequently cleaved into separate mRNAs by *trans*-splicing. Following pre-mRNA processing, the regulation of mature mRNA stability is a tightly controlled cellular process. While many stage-specific transcripts have been identified, previous studies using RNA-seq suggest that changes in overall transcript level do not necessarily reflect the abundance of the corresponding protein.

**Methods**: To better understand the regulation of gene expression in *T. brucei*, we performed a bioinformatic analysis of RNA-seq on total, sub-polysomal, and polysomal mRNA samples. We further cross-referenced our dataset with a previously published proteomics dataset to identify new protein coding sequences.

**Results**: Our analyses showed that several long non-coding RNAs are more abundant in the sub-polysome samples, which possibly implicates them in regulating cellular differentiation in *T. brucei*. We also improved the annotation of the *T.brucei* genome by identifying new putative protein coding transcripts that were confirmed by mass spectrometry data.

**Conclusions**: Several long non-coding RNAs are more abundant in the sub-polysome cellular fractions and might pay a role in the regulation of gene expression. We hope that these data will be of wide general interest, as well as being of specific value to researchers studying gene regulation expression and life stage transitions in *T. brucei*.

## Introduction

*Trypanosoma brucei*, a protozoan parasite transmitted by the tsetse fly, causes human African trypanosomiasis (HAT) and nagana in cattle ^1^. The parasite undergoes a complex lifecycle between its insect vectors and mammalian hosts ^2^: Slender bloodstream form (BSF) parasite proliferate predominantly in the blood and lymph of the infected mammalian host in the first stage of the disease and the second neurological stage of the disease occurs when these parasites cross the blood-brain barrier. Some of the slender BSF parasites differentiate into non-replicative stumpy forms in the bloodstream and these are pre-adapted for transformation into replicating procyclic form in the testse vector midgut. Procyclic forms further differentiate into replicating epimastigote and then non-dividing metacyclic trypomastigote forms during parasite migration to the tsetse salivary glands. The metacyclic parasites are transferred to a new host during a bloodmeal and after differentiation into slender BSF parasites, the lifecycle in complete. The BSF and PCF parasites are the easiest to propagate in the laboratory and are the most studied. 

Transcription is particularly interesting in *T. brucei* because the arrangement of its genes is polycistronic. Thus, RNA Polymerase II (RNA Pol II) transcribes protein-coding genes into large polycistrons containing several transcripts. However, the polycistron does not linger as it is co-transcriptionally processed into individual mRNAs ^3^. The processing of the transcription unit occurs by trans-splicing coupled to cleavage of the 3´ end by the polyadenylation machinery for poly(A) addition ^4, 5^. During trans-splicing, a capped 39-nucleotide (nt) spliced leader (SL) mini-exon is added to the 5′ termini of mRNAs. The SL sequence was first discovered when two different VSG transcripts were found with an identical leader sequence at their 5′ ends, which was not evident in their genomic sequence ^6– 8^. This mini-exon is independently transcribed from a tandem array of 140-nt spliced leader (SL) RNA genes ^9, 10^.

Recent studies using RNA-seq have greatly improved our understanding of the *T. brucei* transcriptional landscape across the BSF and PCF life stages ^2, 11– 15^. These studies have found new transcripts, many non-coding RNAs, and facilitated the correction of numerous annotations across the *T. brucei* genome. While several aspects of translational control have been investigated in *T. brucei*, there are only a few examples of polysome profile analysis that have explored the efficiency of translation between BSF and PCF parasites ^12, 16^. Numerous 80S ribosomes can be translating an mRNA transcript at the same time, producing so-called ‘polysomes’ ^17^. The number of ribosomes on an mRNA generally reflects that transcript’s rate of translation under given conditions ^18^. Further, a particular mRNA’s higher or lower than average association with ribosomes indicates the potential involvement of gene-specific regulatory mechanisms ^19^.

To make a contribution to our understanding of the regulation of gene expression in trypanosomes, we investigated mRNA recruitment to ribosomes with RNA-seq of total polyA+, sub-polysomal, and poly-ribosomal mRNA purified from BSF and PCF life stages of *T. brucei*.

## Methods

### Cell culture

*T. brucei* bloodstream form cells, strain 427, VSG variant MITat1.2 ^20^ (kindly provided by Prof. George Cross) were cultured at 37°C with 5% CO _2_ in cell culture flasks with filter lids (Greiner). Cells were grown to a maximum density of 3x10 ^6^ cells/ml in HMI-9T medium (HMI-9 powder, Gibco Catalog Number: 07490915N). HMI-9T contains variations on the HMI-9 medium described in 21: thioglycerol (Sigma, Catalog Number: m6145) was used instead of β-mercaptoethanol, and GlutaMAX (Gibco, Catalog Number: 35050-38) was used instead of L-glutamine for their increased stability. *T. brucei* procyclic form transgenic cell line 29.13.6 (kindly provided by Prof. George Cross) was cultured at 28°C in Becton Dickinson culture flasks. Cells were grown to a maximum density of 4x10 ^7^ cells/ml in SDM-79 medium (Invitrogen, custom made on request, Catalog Number: N/A) ^22^ supplemented with 15% fetal bovine serum (FBS) (PAA, Catalog Number: A11-101), GlutaMAX (Gibco, Catalog Number: 35050-38), and 15 µg/ml hemin (Sigma, Catalog Number: H9039).

### Polysome fractionation and RNA extraction

Log-phase cultures of *T. brucei* BSF and PCF cells were incubated with 50 µg/ml cycloheximide (Sigma, Catalog Number: C4859) for 10 min prior to the start of polysome purification procedures. Cells were pelleted by centrifugation at 800 g for 10 min at 4°C. PCF cells were washed with PBS (137 mM NaCl, VWR Catalog Number: X190; 2.7 mM KCl, VWR Catalog Number: ICNA0215194401; 10 mM Na _2_HPO _4, _VWR Catalog Number: 4062-01; 2 mM KH _2_PO _4 _pH 7.4, VWR Catalog Number: 26925.295) containing 1 mg/ml cycloheximide (Sigma Catalog Number: C4859), while BSF cells were washed with trypanosome dilution buffer (5 mM KCl, VWR Catalog Number: ICNA0215194401; 80 mM NaCl, VWR Catalog Number: X190; 1 mM MgSO _4 _VWR Catalog Number: 2506-01; 20 mM Na _2_HPO _4, _VWR Catalog Number: 4062-01; 2 mM NaH _2_PO _4, _VWR Catalog Number: ICNA0219550091; 20 mM glucose pH 7.4 _, _VWR Catalog Number: 1910-05) containing 1 mg/ml cycloheximide (Sigma Catalog Number: C4859). Cells were resuspended in polysome lysis buffer (120 mM KCl, VWR Catalog Number: ICNA0215194401; 2 mM MgCl _2 _VWR Catalog Number: ICNA0520984480; 20 mM Tris-HCl pH 7.5 VWR Catalog Number: ICNA04816100; 1 mM DTT Sigma Catalog Number: 10708984001; 1% n-octylglycoside Sigma Catalog Number: 10634425001; 50 µl RNAsin Promega Catalog Number: N2111; 2 µg/ml leupeptin Sigma Catalog Number: L2884; 1 µg/ml aprotinin Sigma Catalog Number: A6279; 1 µM TLCK Sigma Catalog Number: 90182; 1 mM PMSF Sigma Catalog Number: 10837091001; 1mg/ml cycloheximide Sigma Catalog Number: C4859). The detergent n-octylglycoside (NOG) was chosen because it does not absorb at 254 nm. The lysates were loaded on top of 10 ml sucrose (Sigma Catalog Number: S0389) gradients (5 increments, 2ml each: 10%–50% sucrose) and centrifuged for 2 h at 38,000 rpm at 4°C in a Beckman ultracentrifuge using a SW41Ti rotor. Gradients were fractionated (0.5 ml fractions) and analysed for nucleic acid content by a Nanodrop spectrophotometer at 254 nm. RNA was purified using RNeasy kits (Qiagen, Catalog Number: 74104) from pooled sub-polysome and poly-ribosomal fractions. Gradient analysis was also performed using a gradient collector (Teledyne) with continuous monitoring at 254 nm. Individual fractions were collected with a Foxy Jr. (Teledyne) fraction collector. Following collection, the RNA from each sample was purified as above and pooled according to the sub-polysomal and polysomal fractions identified in the absorbance trace.

Total RNA was extracted from bloodstream and procyclic form *T. brucei* using the RNeasy Mini Extraction Kit (Qiagen, Catalog Number: 74104). The protocol was carried out according to manufacturer’s instructions with a few deviations for *T. brucei*. Cells were centrifuged for 10 min, 800 x *g* at room temperature, media was aspirated and the cell pellets were resuspended in buffer RLT (Qiagen, Catalog Number: 79216) and β-mercaptoethanol (Sigma Catalog Number: 444203) was added at a 1:100 dilution. One volume of 70% ethanol (Sigma Catalog Number: 51976) was added to the lysate and the mixture was transferred to the provided column. RNA was bound to the column by centrifugation for 15 sec, 10,000 x *g*. The column was then washed with Buffer RWI and twice with Buffer RPE (Qiagen, Catalog Number: 1018013). Following the washes, the column was transferred to a sterile (RNAse free) Eppendorf tube (Thermofisher, Catalog Number: AM12400), and the RNA was eluted in 50 μl RNase-free H _2_O (Thermofisher, Catalog Number: AM9916). The RNA concentration was then estimated from the A _260_ value using a Nanodrop 2000c spectrophotometer (Thermo) with path length settings adjusted for RNA (40). Following quantitation, the purified RNA was subsequently used for RNA-seq cDNA library preparation.

### Preparation of cDNA libraries for RNA-seq

Total RNA, sub-polysomal, and poly-ribosomal RNA was isolated from BSF and PCF *T. brucei* followed by poly(A) mRNA enrichment with poly-T oligomers attached magnetic beads (Illumina). The mRNA was then fragmented into 200 nt fragments using Covaris Adaptive Focused Acoustics process with the following operating conditions: Sample volume 130 µl, duty cycle 10%, intensity 5, cycles per burst 200, processing time 60 s, water bath temperature 4°C, power mode frequency sweeping, degassing mode continuous. Fragmented mRNA was concentrated by ethanol precipitation and measured on an RNA Pico chip (Agilent 2100 Bioanalyzer). The first strand of cDNA was synthesized using reverse transcriptase (Invitrogen Life Technologies, Catalog Number: 18064-022) and random primers (Invitrogen Life Technologies, Catalog Number: 1880007) using a Omnigene thermal cycler (25°C for 10 min. 42°C for 50 min, 70°C for 15 min), followed by second strand cDNA synthesis using a Omnigene thermal cycler ( 16°C for 60 min), producing double-stranded cDNA (NEBNext mRNA library kit for Illumina, NEB, Catalog Number: E6100. To blunt-end the DNA fragments, an end repair reaction was performed with Klenow polymerase (NEB, Catalog Number: M0210L), T4 DNA polymerase (NEB, Catalog Number: M0203L), and T4 polynucleotide kinase (NEB, Catalog Number: M0201L). A single 3´ adenosine overhang was added to the cDNA allowing the ligation of Illumina adaptors. These adaptors contain primer sites both for sequencing and complimentary annealing onto the Illumina flow cell surface (Top adapter: 5′-ACACTCTTTCCCTACACGACGCTCTTCCGATCT-3’ Bottom adapter 5′-GATCGGAAGAGCGGTTCAGCAGGAATGCCGAG-3’). Adaptor ligated cDNA fragments were measured on an Agilent DNA chip. The final cDNA libraries were sequenced on a HiSeq2000 (Illumina).

### Bioinformatic analysis

The software versions of the packages used for the bioinformatic analysis are listed in the file named “package_versions.txt” and deposited in the zenodo repository mtinti/polysome. The FASTQ files of technical replicates were concatenated together. The forward and reverse paired-end reads of the biological replicates (B_tot: 1 to 3, B_pol: 1 to 3, B_sub: 1 to 3, P_tot: 1 to 3, P_pol: 1 to 3, P_sub: 1 to 3, where B=BSF, P=PCF, tot=total, pol=polysomal, sub=sub-polysomal) were aligned to the reference genome v46 of *T. brucei* clone TREU927 and 427_2018 downloaded from TriTrypDB ^23^ using Bowtie2 ^24^, with the ‘very-sensitive-local’ pre-set alignment option. The alignments were converted to BAM format, reference sorted and indexed with SAMtools ^25^. The genome coverage of the aligned reads was extracted from the BAM files using bedtools ^26^ with the -bg option to output bedGraph files. Fragment counts were determined from the BAM files using featureCounts ^27^ with parameters: -p (pair end) -B (both ends successfully aligned) -C (skip fragments that have their two ends aligned to different chromosome) -M (count multi-mapping) -O (match overlapping features) -t transcript (count level) -g gene_id (summarization level).

### Assembly of Poly A and Spliced Leader Tracks

Alignments with properly paired reads were extracted with SAMtool view using the -f 2 option and parsed with a custom python script to extract the paired reads containing the last 14 bases of the spliced leader sequence (GTGAGGCCTCGCGA) in forward or reverse complement orientation. We used the last 14 bases as they are unique ^28^. The same script was used to extract reads containing poly(A) tracts of at least 10 bases that are often found at the intergenic regions of *T. brucei* ^29^. The aligned reads were saved in BAM format and used to create genomic track coverage in bedGraph format.

### Assembling *T. brucei* transcripts

The GFF annotation file for v46 of *T. brucei* clone TREU927 was downloaded from TriTrypDB and converted to GTF with gffread ^30^. The gene annotation file was supplemented with a recent prediction of long non-coding RNAs ^31^ (doi: https://doi.org/10.1101/2020.05.03.074625). Hypothetical new transcripts were predicted using Trinity ^32^ and Scallop ^33^. First, we identified new predicted genes with Scallop that was run for each biological replicate. The scallop predictions in GTF format were filtered to include only genes in intergenic regions that did not have any overlap with previously annotated genes. To achieve this, the GTF prediction files and the GTF reference file were converted to bed format with gtf2bed and intersected using bedops ^34^. The filtered regions were converted back to GTF format, merged in a set of unique prediction with StringTie ^35^ and added to the reference GTF file. In a second run, we used Trinity that was executed with the genome guided and jaccard clip parameters for each biological replicate. The predicted Trinity gene sequences were aligned to the TREU927 genome with gmap ^36^ and the GFF output files of gmap were converted to GTF with gffread ^30^. From this point, the same filtering methods used for the Scallop predictions were applied to the Trinity predictions that were added to the reference GTF file. We also downloaded from GenBank ^37^ the genomic sequences and GFF annotation files for the entries: M94286 (maxicircle sequences), FM162566 427 VSG bloodstream form expression site 1 (BES1) locus, FM162567 427 BES2 locus and the minicircle sequences L25588, L25589, L25590, M15321. The GFF downloaded from GenBank were converted to GTF files with Biopython ^38^. We also constructed a synthetic chromosome of VSG 427 gene transcripts with the sequences deposited at http://tryps.rockefeller.edu/ using the link http://129.85.245.250/Downloads/vsgs_tb427_all_atleast150aas_cds.txt. The VSG sequences were concatenated with random DNA sequences of 50 base pairs to produce the synthetic chromosome (named fake_vsgs) and a GTF annotation file was produced. All the GTF annotation files were concatenated together as well as the gene sequences to produce a new assembly named tb927_5 (tb927_5.gtf).

### Quality control

The quality of alignments were evaluated with Qualimap2 ^39^ using the bamqc and rnaseq options. The Qualimap2 output files, and the outputs of fastp, bowtie2, Picard Mark Duplicates, SAMtools flagastat, SAMtools stats and featureCounts were aggregated with MultiQC ^40^, inspected and made available at https://polysome-qc.onrender.com. Dimensionality reduction was performed with the MDS algorithm implemented in SciPy ^41^ after log2 transform of the read counts of the top 500 expressed gene. The length and GC content of the predicted transcripts were extracted using bedtools nuc function after converting the GTF annotation file to bed format. The GC and length content biases were assessed with the cqn package for R ^42^ after removing genes with low counts using edgeR ^43^. RPKM values for the dataset visualization were extracted using the cqn package for R.

### Dataset visualization

Zero counts were replaced by the minimum value counts column-wise. The ANOVA-like test in edgeR was used to retain genes that differ in abundance in at least one of the samples with a false discovery rate <1%.

### RadViz

The RadViz function implemented in the pandas python library ^44^ was modified and used for the visualization. For each gene the median value of the three biological replicates was computed for each experiment (B_tot: 1 to 3, B_pol: 1 to 3, B_sub: 1 to 3, P_tot: 1 to 3, P_pol: 1 to 3, P_sub: 1 to 3). For visualization, each gene was colour coded and assigned to one of the six experiments (B_tot, B_pol, B_sub, P_tot, P_pol, P_sub) where it showed the maximum abundance value.

### Clustering

The dataset was normalized raw-wise with a standard scale approach, by subtracting the minimum value and dividing by the maximum value minus the minimum value, for each gene count. The optimal number of clusters was determined with the elbow approach using the KElbowVisualizer function implemented in the yellobrick python package ^45^. The dataset was divided in 4 clusters using the K-means algorithm implemented in the scikit-learn python package ^46^.

### lncRNAs enrichment

The first spreadsheet “Ksplice lncRNAs” in Supplemental Table 1 of doi: https://doi.org/10.1101/2020.05.03.074625 ^31^ was used to extract the hypothetical long non-coding mRNAs. The hypergeometric test implemented in scipy stats ^41^ was used to compute the enrichment p-value for long non-coding genes in each cluster.

### mRNA half-life

The “BS mRNA half-life (min)” and “PC mRNA half-life (min)” columns from Table S5 of Antwi *et al*., 2016 ^12^ were used to extract the mRNA half-lives. The gene IDs were converted to those of version 46 of the TREU927 genome using TryTripDB.

### GO term enrichment

The GO enrichment analysis was performed with the goatools python package ^47^. The go-basic.obo file was downloaded with the goatools python package. The gaf association file was downloaded from TritrypDB. Enriched go term p-values were corrected with the Bonferroni option in goatools and filtered at 1% false discovery rate. For visualization, the GO terms were further filtered to include terms appearing uniquely in one of the clusters. The enriched GO terms in each cluster were sorted according to the adjusted p-value and the top-5 GO terms retained.

### Identification of new protein coding genes

The Raw files described in our protein half-lives paper ^48^ were processed in MaxQuant with the same parameters used to compute the iBAQ values, except that the predicted amino acidic sequences from the open reading frames downloaded from TriTrypDB version 46 were used. The start and end coordinates of the identified peptides were retrieved from the peptides.txt output files and organized in bed format. The coverage values of the genomic peptide coordinates in the bed file were set to 1. The file was sorted with the sort-bed function in bedtools. We then extracted the new gene predictions from our assembled GTF file and converted them to bed format. Subsequently, we used the bedextract function in bedtools to extract the peptides mapped to new predicted transcripts. The web interface of the phobius program ^49^ was used to search for transmembrane domains and the web interfece ot the signalP algoritms 3.1 and 5.0 ^50^ were used to search for signal peptides. The blast ^51^ searches were performed with the web interfaces implemented at the NCBI or TriTrypDB. The Clustal Omega analysis were performed with the web interfaces implemented at EMBL-EBI ^52^.

***Coverage Visualisation.*** The software versions of the packages used for the visualisation of the bedGraph files are listed in the file named “package_versions.txt” and deposited in the zenodo repository mtinti/polysome_coverage. The bedGraph files were visualized with the svist4get python package ^53^.

### Comparison with previous work

***Transcription competency.*** Table S5 from Antwi *et al*., 2016 ^12^ was downloaded and the Ribosomes/kb on polysomes values were extracted from spreadsheet 1 (PCF) and spreadsheet 2 (BSF). Gene names were mapped to the version 46 of TREU927 genome using the gene search service at TriTrypDB ^23^. Fragment counts for our dataset were determined from the BAM files using featureCounts ^27^ with parameters: -p -B -C -M -T 8 -t CDS -f to count only reads mapped to CDS regions. The read counts were filtered for low counts and normalized using edgeR ^43^. Before computing the fraction of transcripts in polysomes, the polysome read counts were divided by 0.7 and the sub-polysome read counts were divided by 0.3 to correct for the total amount of mRNA found in polysome (70%) and sub-polysomal fractions (30%) ^12^. The median of the fraction of transcripts in polysomal fraction was computed for the three biological replicates of BSF and PCF life stages and compared to the values reported in Antwi *et al*., 2016 ^12^. The Pearson correlation coefficients between samples were computed with the python package pandas ^41^.

### Ribosome profile

The fastq files for the ribosome profile experiment were downloaded from the ENA archive ^54^ with accession number PRJEB4801 and processed in a similar way as reported in Vasquex *et al.* 2014 ^2^. Briefly, the fastq files for the BSF and PCF biological replicates samples were concatenated together and the Illumina adaptor sequences were trimmed with the fastp package ^55^. Sequences shorter than 20 bases were removed with the fastp package ^55^. Reads were aligned, counted, and normalized as described above. The aligned reads in BAM format were used to create genomic track coverage in bedGraph format.

### Sub-polysome / polysome differential abundance analysis and Grumpy Like Genes

Differential abundance analyses were carried out with edgeR using generalized linear models (GLM) and the correction factors provided by the cqn package. In this study, we tested the differential abundance between the sub-polysome and polysome samples of the BSF and PCF life stages. To identify the Grumpy Like genes ^31^ we created a third model to study the differential transcript abundance between the sub-polysomal samples (mixed model of BSF and PCF) against the sub-polysomal samples (mixed model of BSF and PCF). The p-values of the test were corrected with the topTags function in R using the Benjamini–Hochberg method.

The code to reproduce the analysis pipeline and the figures, the raw data and additional python scripts used for this study are available at GitHub.

## Results

**Figure 1.**
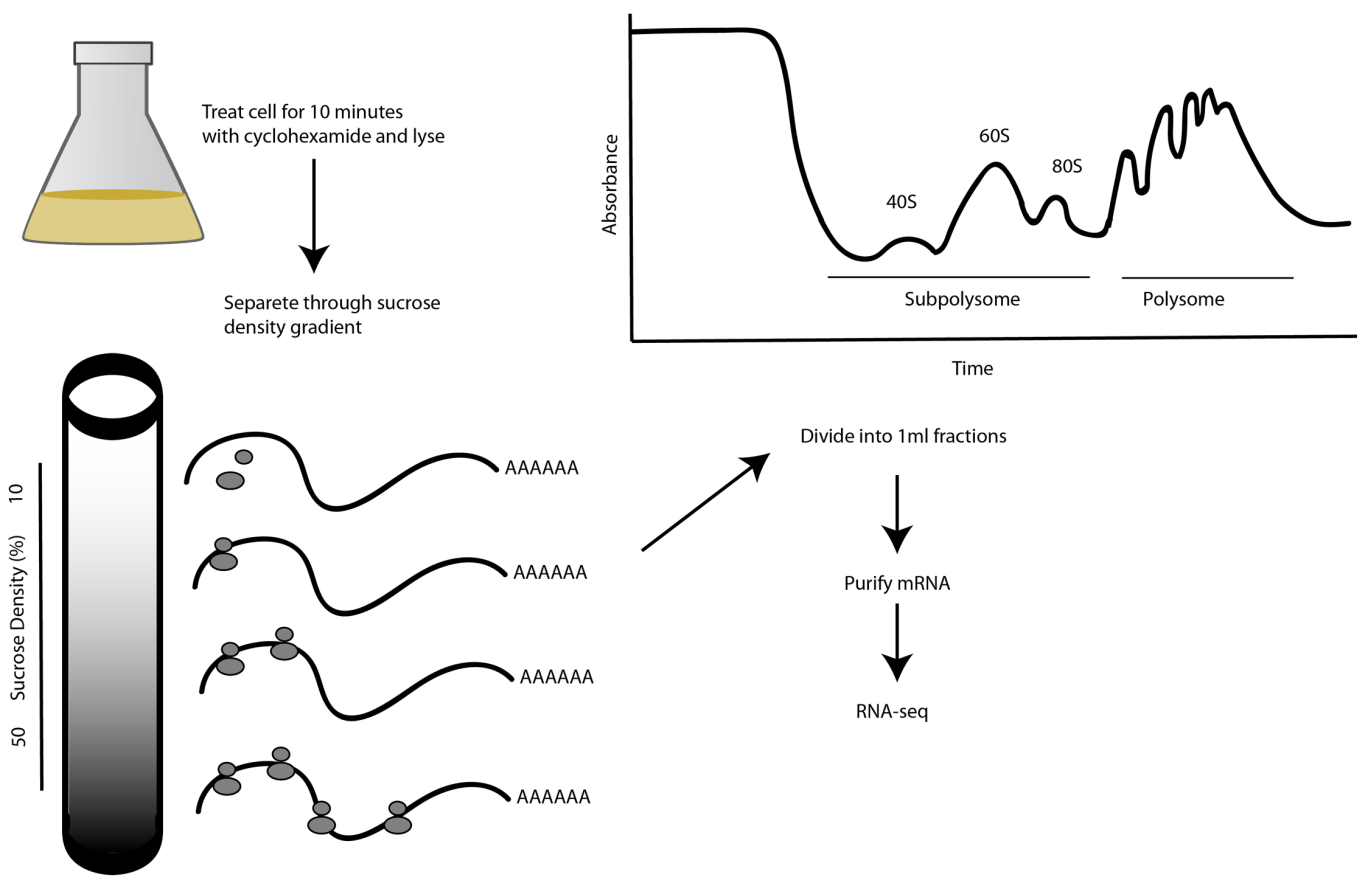
Experimental design. Cycloheximide-treated cells are lysed by detergent and their contents separated by centrifugation through a sucrose gradient. A representative 254 nanometer (nm) absorbance trace for nucleic acids in a Bloodstream Form (BSF) lysate density gradient is shown, normalized to the absorbance of a blank gradient. The earliest fractions contain the sub-polysomal fraction and the latest fractions contain the polysomal fraction. Free monosomes (80S) and ribosomal subunits (40S and 60S) are indicated with arrows. The messenger RNA (mRNA) transcripts from total, sub-polysomal and polysomal RNA were purified on immobilized oligo-dT for RNA sequencing (RNA-seq).

In our study, cells were treated with the antibiotic cycloheximide to prevent polysome run-off during sample preparation. Cycloheximide binds to the 60S ribosomal subunit and arrests translation elongation by inhibiting release of the deacylated tRNA from the ribosome E site, thereby stalling the ribosomes on mRNA in a polysomal state ^56^. The high protein content of polysomes allows them to be separated throughout a sucrose gradient according to the number of ribosomes attached to the mRNA ( Figure 1). To prepare samples for RNA-seq, cDNA libraries were generated from both total mRNA, polysome-associated mRNA and sub-polysomal mRNA transcripts. It is important to note that our procedure enabled the libraries to be completed without PCR amplification, therefore eliminating sample bias associated with variable amplification. In all, three (1 to 3) biological and three technical replicates of total (tot), sub-polysomal (sub), and polysomal (pol) mRNA RNA-seq experiments were performed for BSF (B) and PCF (P) life stages.

### Assembling a reference transcriptome

Whole transcriptome experiments offer valuable resources to detect new genes and improve gene models. For this reason, we decided to create a complete TREU927 transcriptome assembly before assigning our reads to the reference gene set. To this end, we first added a set of newly predicted genes described by Guegan, *et al.* ^31^ encoding mostly long non-coding RNA (lncRNA). Subsequently, we implemented a genome-guided approach to annotating new genes discovered from our dataset. This strategy consisted of mapping reads along the reference genome, followed by gene prediction (Methods). This final step allowed us to extend the number of transcribed genome loci from 11725 to 15743 (an increase of 34%).

To aid the visualization of the newly predicted genes and assess the quality of the transcript boundaries, we extracted from all the samples the reads containing a spliced leader sequence and poly(A) genomic tract of >9 bases. The spliced leader sequence is present at the beginning of all mature trypanosome transcripts and can be used to determine the exact 5’ boundary of the gene. The poly(A) genomic tracts are often present in intergenic regions and can help to determine the 3’ gene boundaries. It is useful to note that the script we used to select the poly(A) genomic tracts also selects reads with poly(A) mRNA tails. However, we did not distinguish between poly(A) mRNA tails or poly(A) genomic tracts as both are useful to define gene boundaries ^57^.

### Quality control

**Figure 2.**
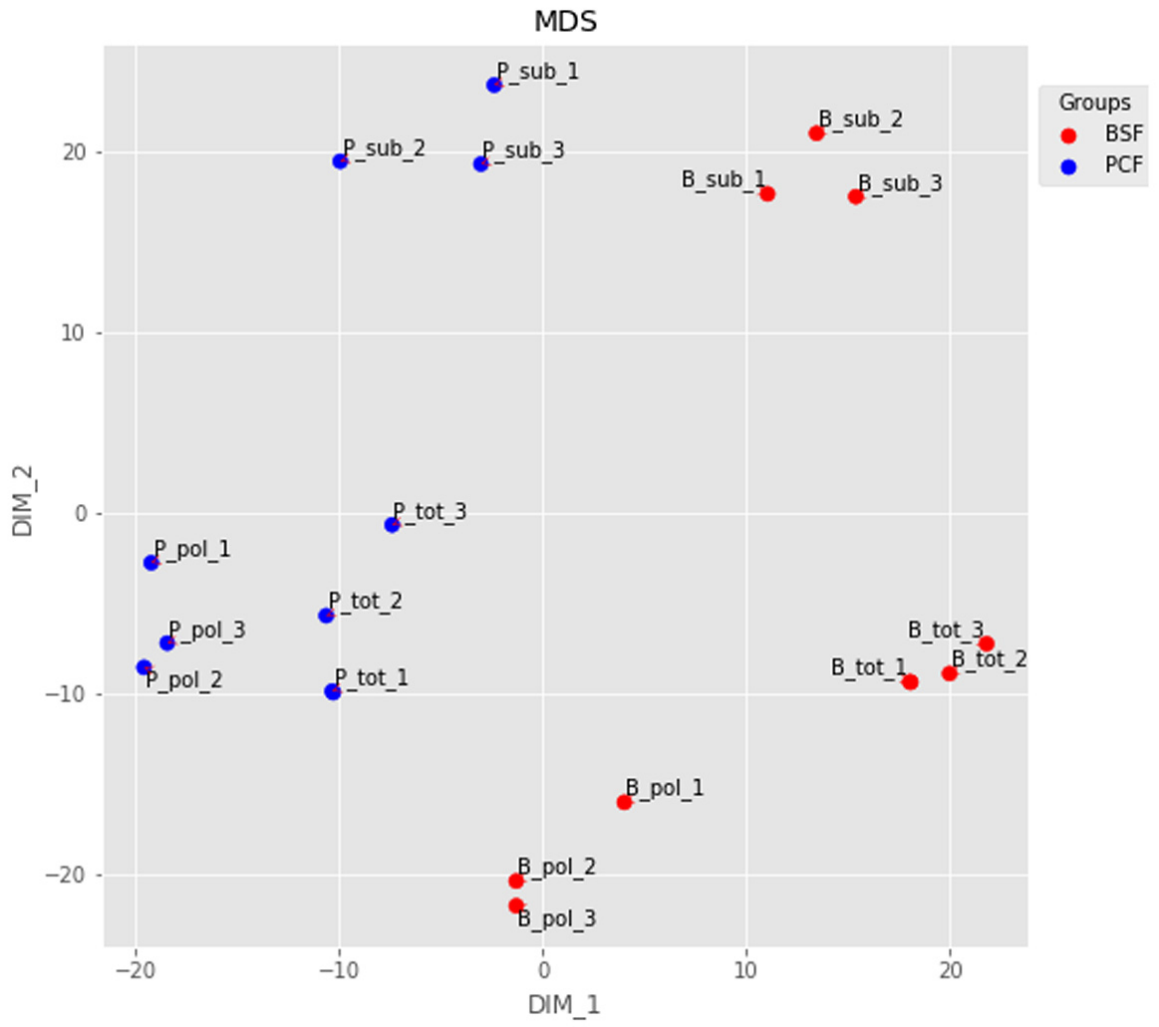
Dimensionality reduction. The output of a multidimensional scaling analysis of the top 500 transcripts for: B_tot_1-3 = Bloodstream Form (BSF) total messenger RNA (mRNA) from samples 1-3; B_sub_1-3 = BSF sub-polysomal mRNA from samples 1-3; B_pol_1-3 = BSF polysomal mRNA from samples 1-3; P_tot_1-3 = Procyclic Form (PCF) total mRNA from samples 1-3; P_sub_1-3 = PCF sub-polysomal mRNA from samples 1-3; P_pol_1-3 = PCF polysomal mRNA from samples 1-3.

**Figure 3.**
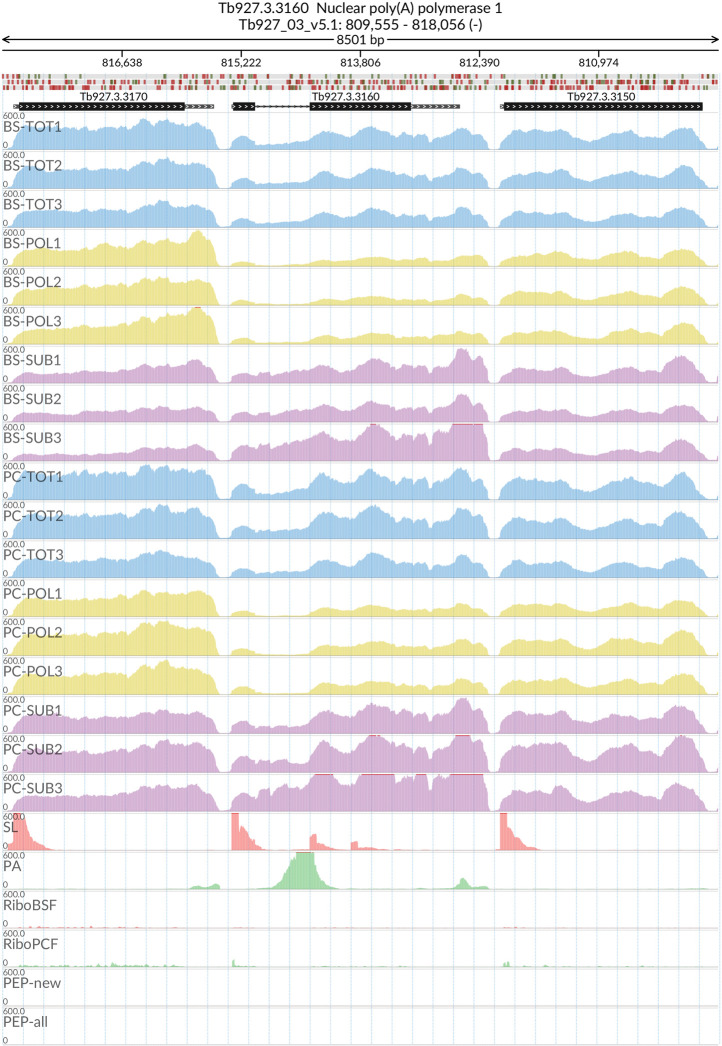
Genome coverage for Tb927.3.3160. For the intron containing gene Tb927.3.3160 (Nuclear poly(A) polymerase 1) the figure shows the genome coverage for the total (TOT), polysomal (POL), and subpolysomal (SUB) samples (biological replicates 1 to 3) of the bloodstream (BS) and procyclic (PC) form life stages. The figure also reports the genome coverage of the Splice Leader (SL) and poly(A) mRNA tails and/or poly(A) genomic tract (PA) containing reads assembled from the samples. Also shown are the ribosome profiling reads for the Bloodstream Form (RiboBSF) and Procyclic Form (RiboPCF) life stages as described in Vasquex *et al*. 2014. The last two genomic tracks report the peptide identifications for new predicted open reading frames (PEP-new) and for all the open reading frames (PEP-all) in TritrypDB. The maximum height of each of the gene tracks is reported on the top left of each track. The top of the figure shows an ideogram of the gene structures. The three grey genomic tracks at the top report ATG codons in green and stop codons in red.

**Figure 4.**
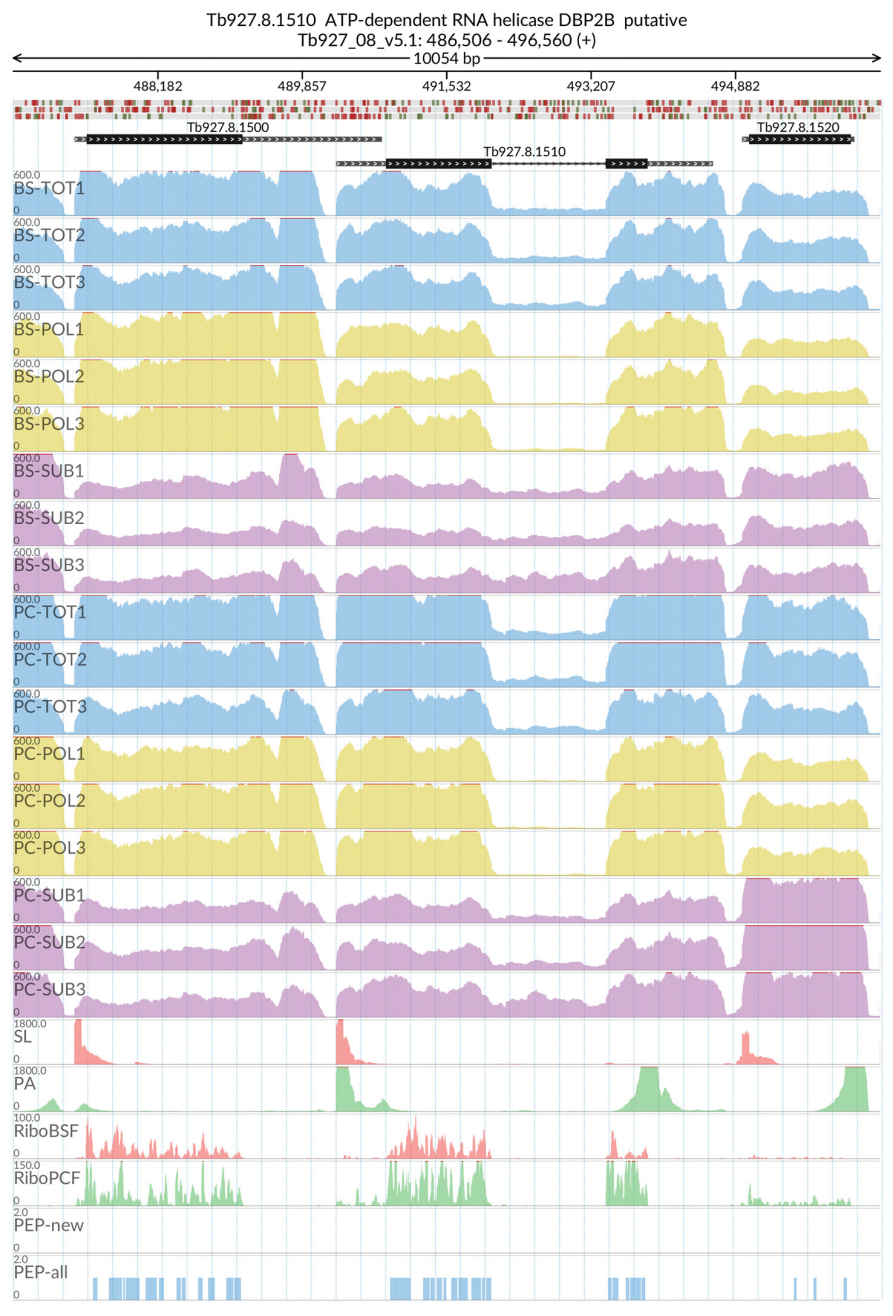
Genome coverage for Tb927.8.1510. For the intron containing gene Tb927.8.1510 (ATP-dependent RNA helicase DBP2B) the figure shows the genome coverage for the total (TOT), polysomal (POL), and subpolysomal (SUB) samples (biological replicates 1 to 3) of the bloodstream (BS) and procyclic (PC) form life stages. The figure also reports the genome coverage of the Splice Leader (SL) and poly(A) mRNA tails and/or poly(A) genomic tract (PA) containing reads assembled from the samples. Also shown are the ribosome profiling reads for the Bloodstream Form (RiboBSF) and Procyclic Form (RiboPCF) life stages as described in Vasquex *et al*. 2014. The last two genomic tracks report the peptide identifications for new predicted open reading frames (PEP-new) and for all the open reading frames (PEP-all) in TritrypDB. The maximum height of each of the gene tracks is reported on the top left of each track. The top of the figure shows an ideogram of the gene structures. The three grey genomic tracks at the top report ATG codons in green and stop codons in red.

The RNA-seq reads were aligned to the TREU927 reference genome, and the numbers of fragments mapping to our assembled gene list were computed. We evaluated the quality of our dataset at several levels. First, we used multidimensional scaling (MDS) to visualise the similarity between the different RNA-seq samples ( Figure 2). The MDS analyses confirmed the high reproducibility of all biological replicates that cluster closer together within each sample type than between sample types. We also evaluated the reliability of our dataset by visualizing the read coverage of the only two known intron-containing genes in the *T. brucei* genome: Tb927.3.3160 (Nuclear poly(A) polymerase 1) and Tb927.8.1510 (ATP-dependent RNA helicase DBP2B). The visualisations in Figures 3 and Figures 4 show that the intron containing regions of the two genes have a sudden drop with little or no coverage in the polysomal samples (yellow tracks) relative to the total and sub-polysomal samples (blue and purple tracks) in both the BSF and PCF samples.

### Comparison with previous work

**Figure 5.**
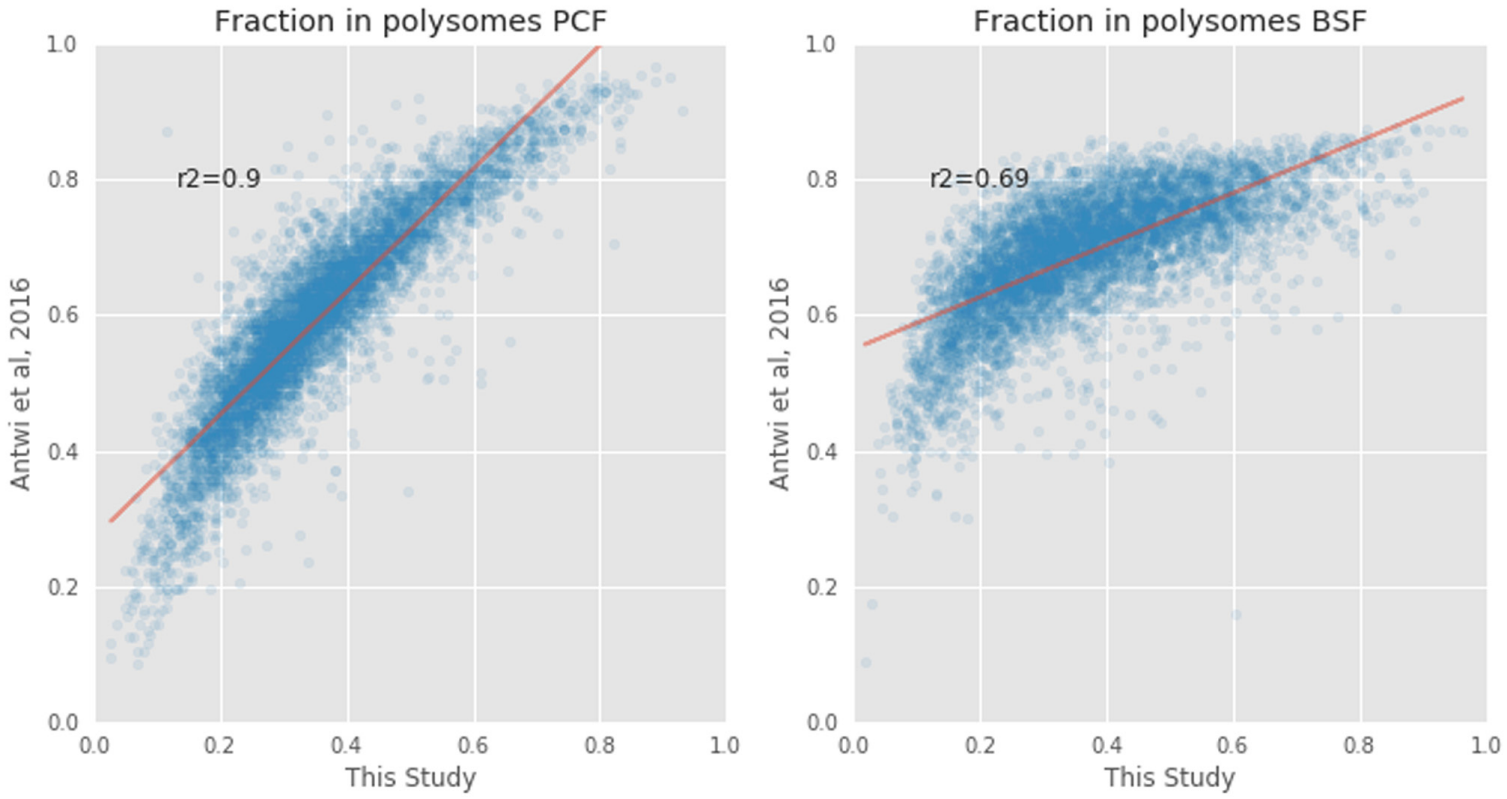
Comparison of the polysomal transcripts between this study and that of Antwi *et al.* ^12^. The proportions of messenger RNA (mRNA) transcripts (blue circles) found in polysomal fractions in 7 (y=axis) and in this study (x-axis) in Procyclic From (PCF, left plot) and Blood Stream From (BSF, right plot) samples. The Pearson correlation coefficients (r ^2^) are 0.90 and 0.71, respectively.

We compared our results with those of Antwi *et al.* ^12^ that describes a similar approach to that used in this study. We first analyzed our dataset by counting reads aligned to coding sequence regions (CDS) only. After read normalization in edgeR, we computed the percentages of transcripts bound by the polysome for each gene. These values were then corrected for the relative proportions of mRNA found in polysome fractions (70%) and sub-polysomal fractions (30%) to mimic the analysis pipeline described in 7 as closely as possible. The percentage of transcripts bound by polysomes from our study was then compared with those reported in Table S1 of 7 ( Figure 5). The comparison showed a stronger correlation in the PCF life stage (R ^2^=0.92) than in the BSF life stage (R ^2^=0.71).

### Bias correction

**Figure 6.**
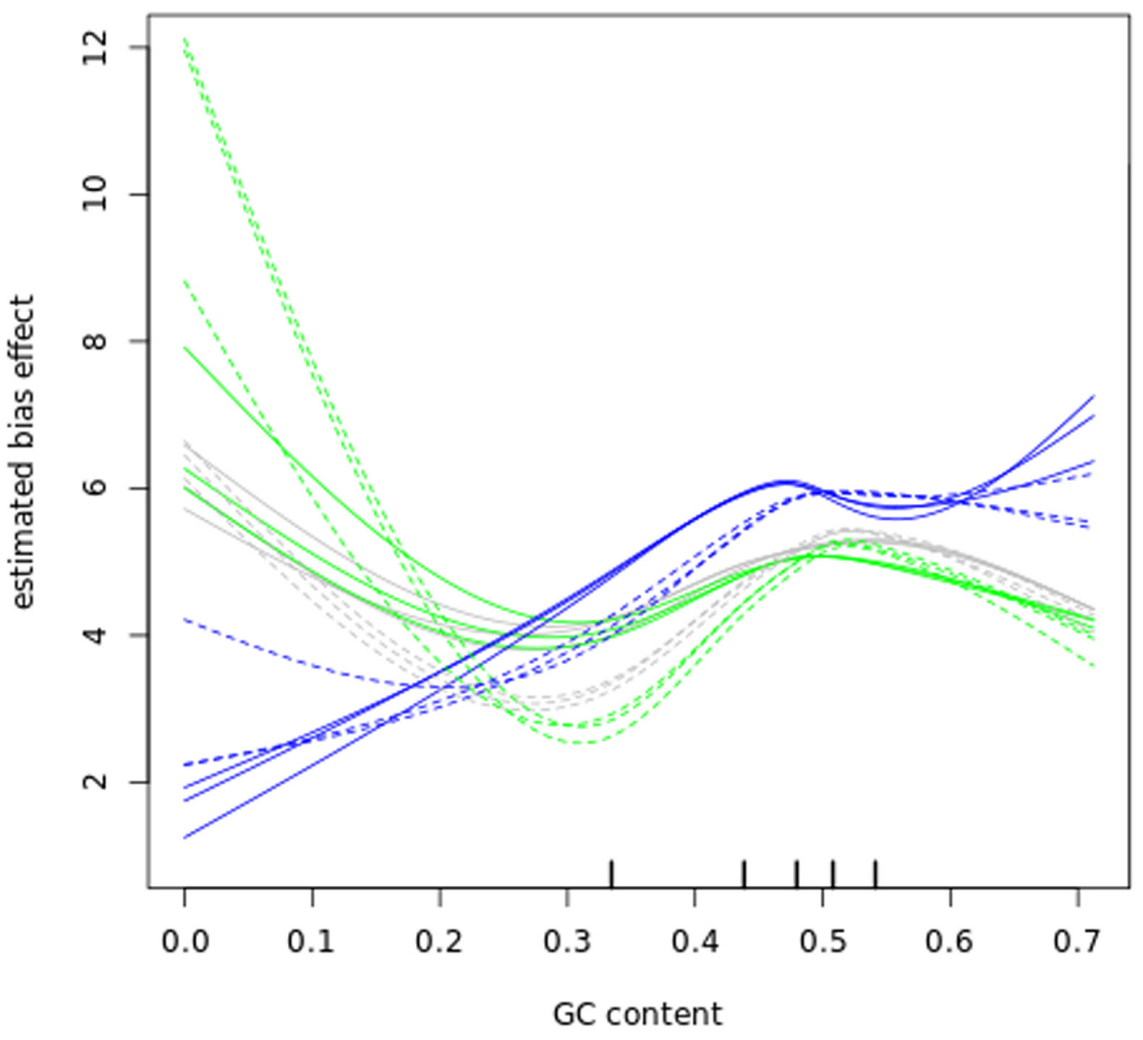
GC bias. A plot of gene transcript guanine-cytosine (GC) content percentage (x-axis) versus the log2 Reads Per Kilobase of transcript, per Million mapped reads (RPKM) estimated bias effect (y-axis) of the bloodstream (B, solid lines) and procyclic (P, dashed lines) samples. The blue lines plot the sub-polysomal (sub) samples, the green lines plot the polysomal (pol) samples and the grey lines plot total (tot) sample bias effects.

**Figure 7.**
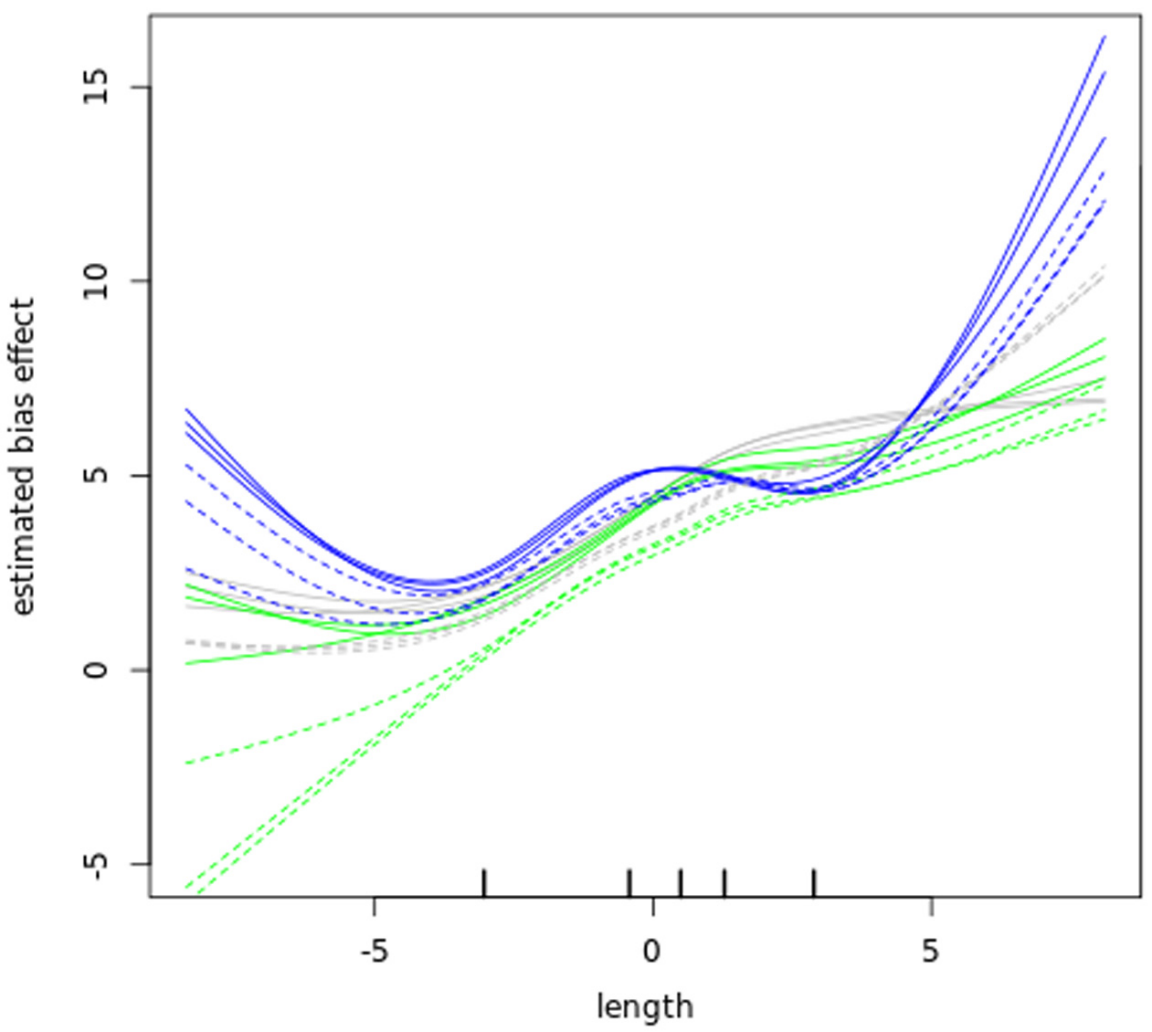
Length bias. A plot of gene transcript length (log2 kilobase) along the x-axis versus the estimated log2 Reads Per Kilobase of transcript, per Million mapped reads (RPKM) bias effect (y-axis) of the bloodstream (B, solid lines) and procyclic (P, dashed lines) samples. The blue lines plot the sub-polysomal (sub) samples, the green lines plot the polysomal (pol) samples and the grey line plots total (tot) sample bias effects.

Before further analysing our datasets, we examined GC content bias and length bias in our read counts as those have been reported to affect RNA-seq experiments ^42, 58, 59^. The data in ( Figure 6 and Figure 7) show that GC content and length biases affect our dataset in a sample-specific way, especially between the sub-polysomal samples (green) relative to the polysomal (blue) and total (grey) samples. We corrected the read counts for these biases and normalized the read counts using the conditional quantile normalization method implemented in the cqn R package ^42^.

### Differential abundance analysis

**Figure 8.**
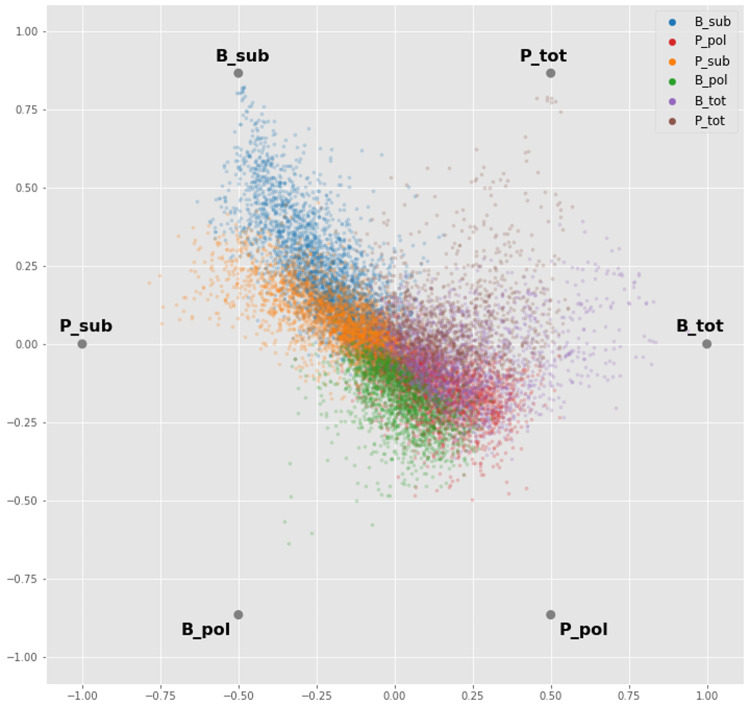
Radial Visualization. A plot from the RadViz algorithm applied to the experimental samples arrayed uniformly around the circumference of a circle. Each gene (dots) is plotted on the interior of the circle such that the distance of the dot on a line from the circumference to the centre is proportional to the gene counts. The dot is colour coded according to the sample where it has the maximum read count value. P = procyclic form, B = bloodstream form, sub = sub-polysomal transcripts, pol = polysomal transcripts, tot = total transcripts.

Before proceeding to the differential abundance analysis, we visualized the whole dataset with a dimensionality reduction technique. Using an ANOVA-like test implemented in edgeR, we found transcripts that are differentially abundant between any of the groups, without biasing before-hand which groups might be different. We then took the median value of each biological replicate for each gene and applied a radial visualization plot that uses a polar coordinate system to visualize the dataset. Sample types are like hours on the clock-face (i.e. related to the angle of the polar coordinate system) and the orthogonal axis (i.e. the distance from the centre) relates to the relative abundance of a gene across the samples. This analysis showed a strong signature for the BSF and PCF sub-polysomal samples, where many transcripts showed the greatest differential abundance relative to all of the other samples ( Figure 8, blue and orange gene dots).

**Figure 9.**
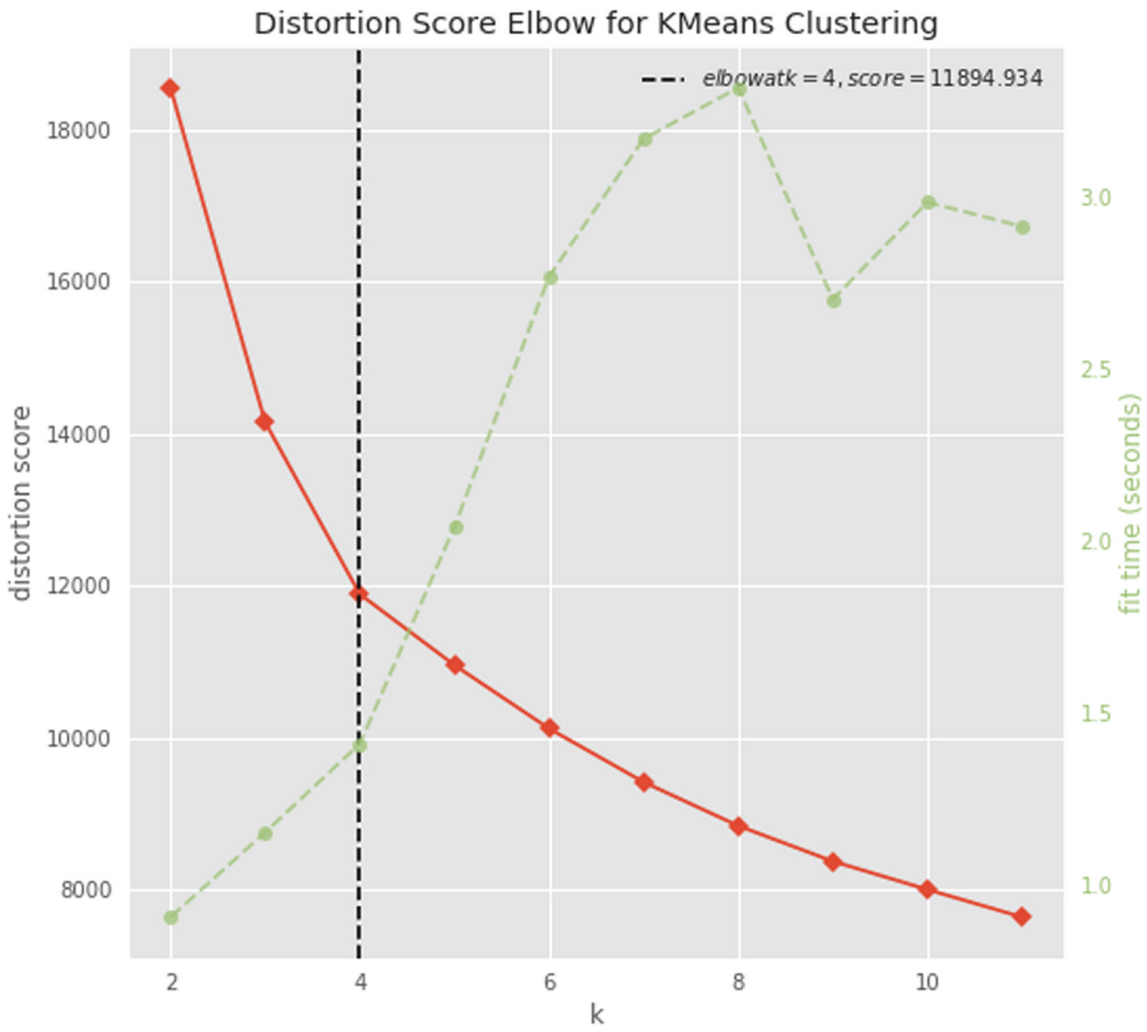
Determining the optimal number of clusters. A plot of the number of clusters tested (K) on the x-axis and the clustering distortion score (the sum of square distances from each point to its assigned cluster center) on the y-axis. The figure also displays the amount of time needed to train the clustering model per K as a dashed green line. If the line chart resembles an arm, then the “elbow” (the point of inflection on the curve) is a good indication that the underlying model fits best at that point ( https://www.scikit-yb.org/en/latest/api/cluster/elbow.html).

**Figure 10.**
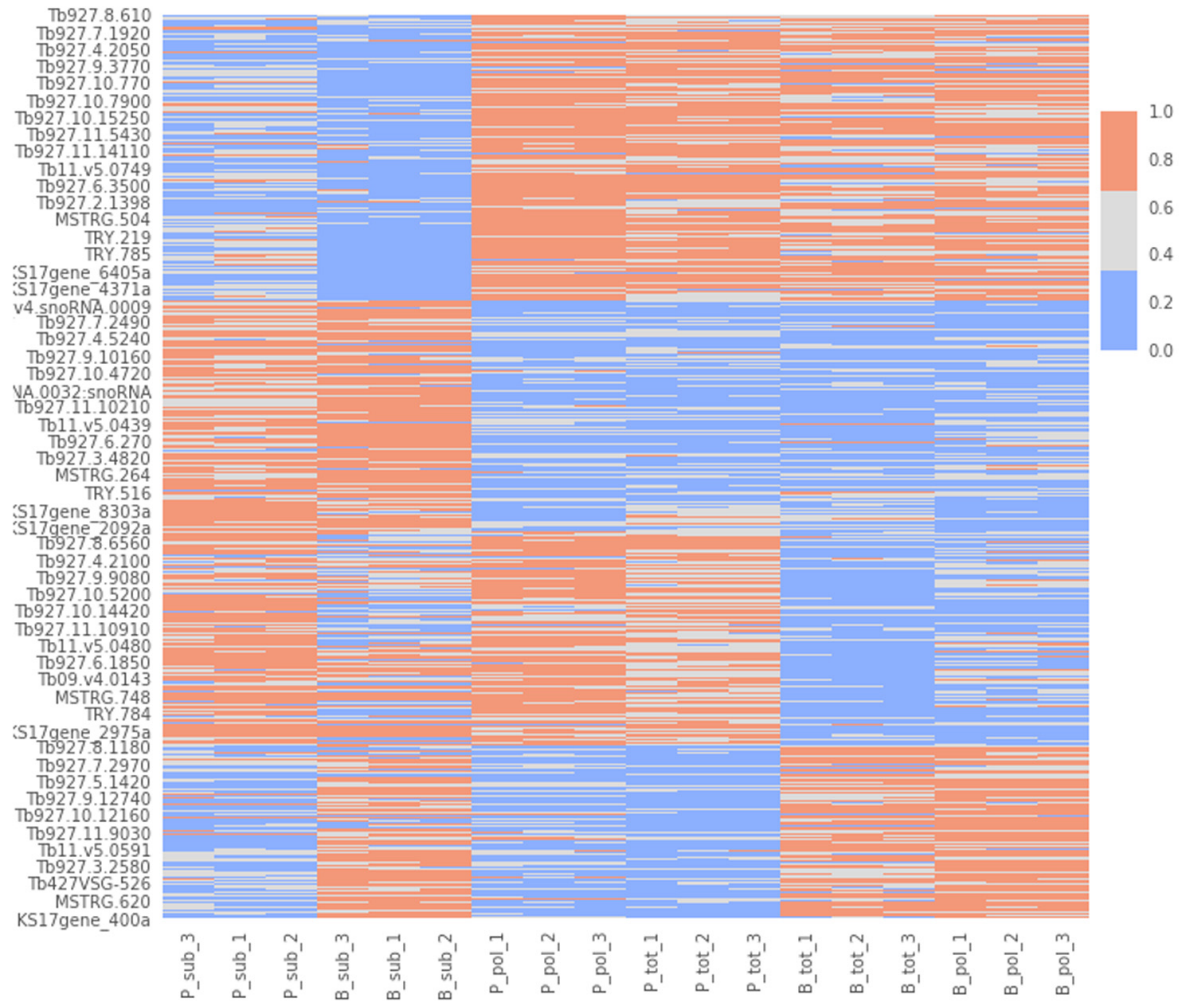
Cluster visualization. A heatmap of the normalized gene count values for the biological replicates (x-axis) against gene identifications (IDs, y-axis). The figure uses three colour codes (colour bar, top right) to visualize the intensity of the normalized read counts (red - highest, gray - middle, blue - lowest). The biological replicates are listed in the format of [B/P]_[tot/pol/sub]_[1/2/3] where B: bloodstream form, P: procyclic form, tot: total RNA sample, pol: polysomal sample, sub: subpolysomal sample, 1,2,3: biological replicate identifiers.

To try to gain insight into this signature, we performed a cluster analysis. We first determined the optimal number of clusters (n=4) with the elbow approach ( Figure 9), and then applied a k-means clustering algorithm to divide our dataset into 4 clusters ( *Extended data*: Table 3 ^60^). Cluster 1: gene transcripts that are more abundant in PCF versus BSF samples. Cluster 2: gene transcripts that are more abundant in BSF and PCF sub-polysomal samples than in all other samples. Cluster 3: gene transcripts that are more abundant in BSF versus PCF samples. Cluster 4: gene transcripts that are less abundant in BSF and PCF sub-polysomal samples than in all other samples. This clustering analysis confirmed the presence of a group of genes (Cluster 2, n= 3356) with the highest read counts in the BSF and PCF sub-polysomal samples relative to all other samples ( Figure 10).

**Figure 11.**
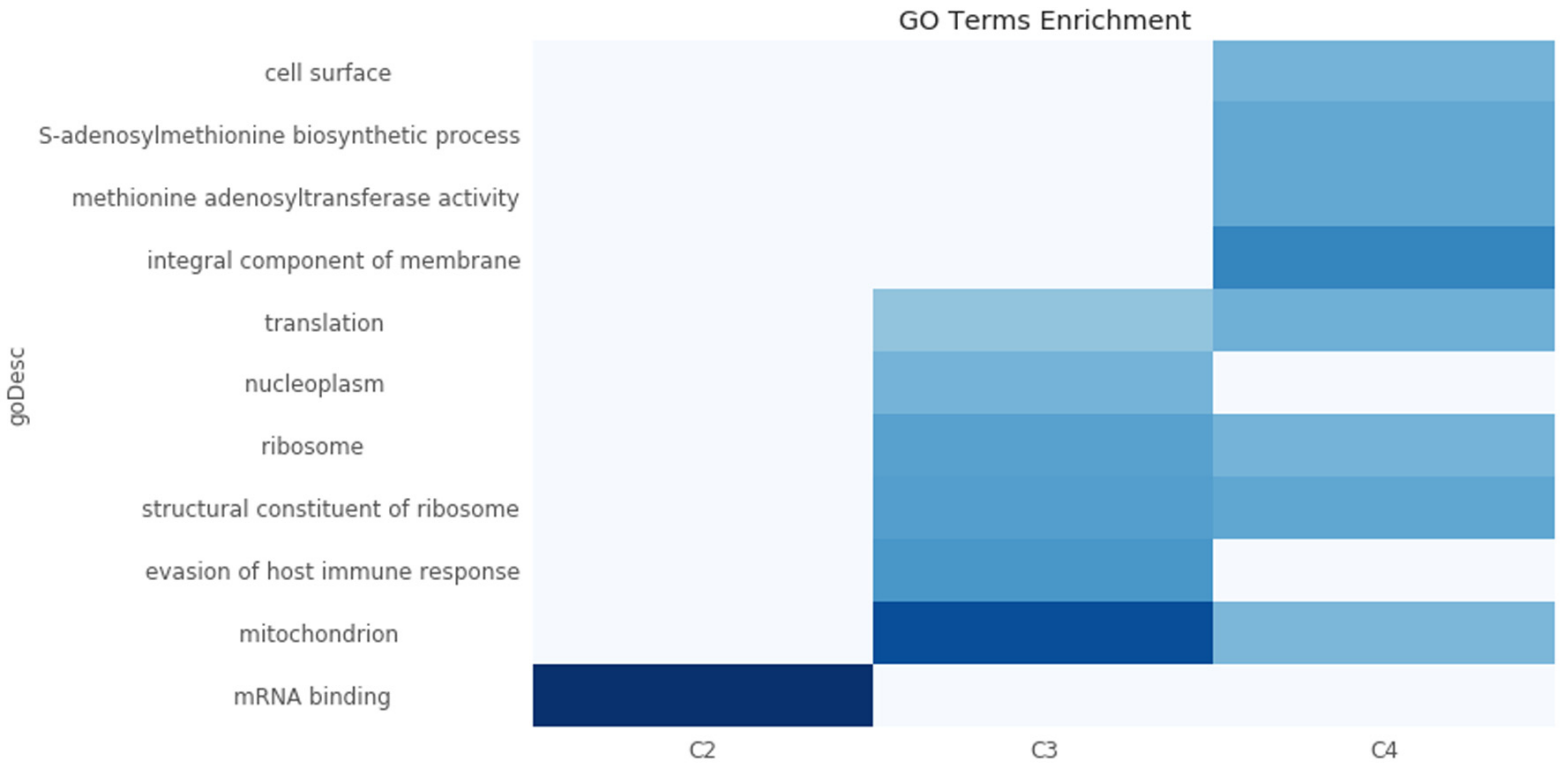
GO term enrichment analysis. A heatmap of the -log10 p-value of the Gene Ontology (GO) term enrichment test. The clusters are plotted in the x-axis and the top enriched GO terms on the y-axis. The -log10 p-value is colour coded according to the colormap on the bottom-right of the plot. The GO terms enriched in >2 clusters have been removed. The cluster C1 (underrepresented in sub-polysomal samples) has been removed from the figure for visualization as it reports the longest list of enriched GO terms (n=41). C2: Cluster 2, genes with the highest gene counts in the Bloodstream From (BSF) and Procyclic Form (PCF) sub-polysomal samples. C3: Cluster 3, genes that are more highly present in BSF samples with respect to PCF samples. C4: Cluster 4, genes with a lower abundance in the sub-polysomal BSF and PCF samples with respect to all the other samples.

**Figure 12.**
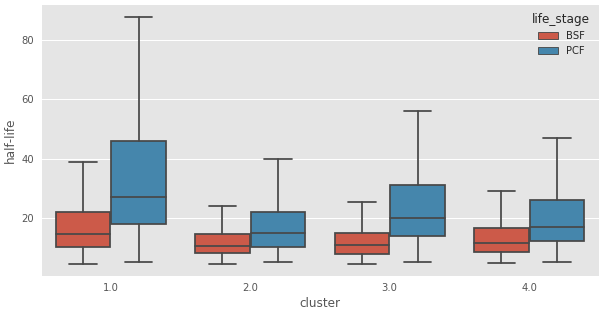
Transcript half-life. Boxplots of messenger RNA (mRNA) half-life in minutes (y-axis) for the genes assigned to the clusters reported in the x-axis for the Bloodstream From (BSF, red) and Procyclic Form (PCF, blue) life stages. 1: Cluster 1, transcripts underrepresented in BSF and PCF sub-polysomal samples, 2: Cluster 2, genes with the highest read counts in the BSF and PCF sub-polysomal samples 3: Cluster 3, genes that are more highly present in BSF samples with respect to PCF samples; 4: Cluster 4, genes with a lower abundance in the sub-polysomal BSF and PCF samples with respect to all the other samples.

To assign possible biological functions to the clusters, we performed a GO-term enrichment analysis across the four clusters. We only retained GO terms that were enriched in at most two of the four clusters, and those with false discovery rates of >1%. This analysis, visualized in ( Figure 11), showed that the transcripts in Cluster 2 (C2) are highly enriched for those encoding mRNA binding proteins. Interestingly, the average half-life of the transcripts in Cluster 2 are the shortest in the BSF and the PCF life stages, when compared to the mRNA half-lives of the transcripts in the other clusters ( Table 1 and Figure 12). We then asked if any of the clusters are particularly enriched for the long non-coding genes identified in 31 and found they are mostly enriched in Cluster 2 ( Table 2). Cluster 2 also has the highest number of two other classes of non-coding mRNAs: the snoRNAs and H/ACA-like snoRNAs ( Table 2).

**Table 1.** Half life report. Median half-lives for each cluster of messenger RNA (mRNAs) as extracted from Antwi *et al*. ^12^.

Cluster	half-life(PCF)	half-life(BSF)
4	27.0	14.3
1	21.0	10.8
2	15.0	10.6
3	17.0	11.6

**Table 2.** Non coding mRNA counts. The number of Small nucleolar RNAs (snoRNAs), H/ACA-like containing box snoRNAs (H/ACA-like snoRNAs ) and long non-coding RNAs (lncRNAs) identified in each cluster.

Cluster	snoRNAs	H/ACA-like snoRNAs	lncRNAs
**1**	17	5	405
**2**	180	43	473
**3**	66	12	330
**4**	20	6	206

**Figure 13.**
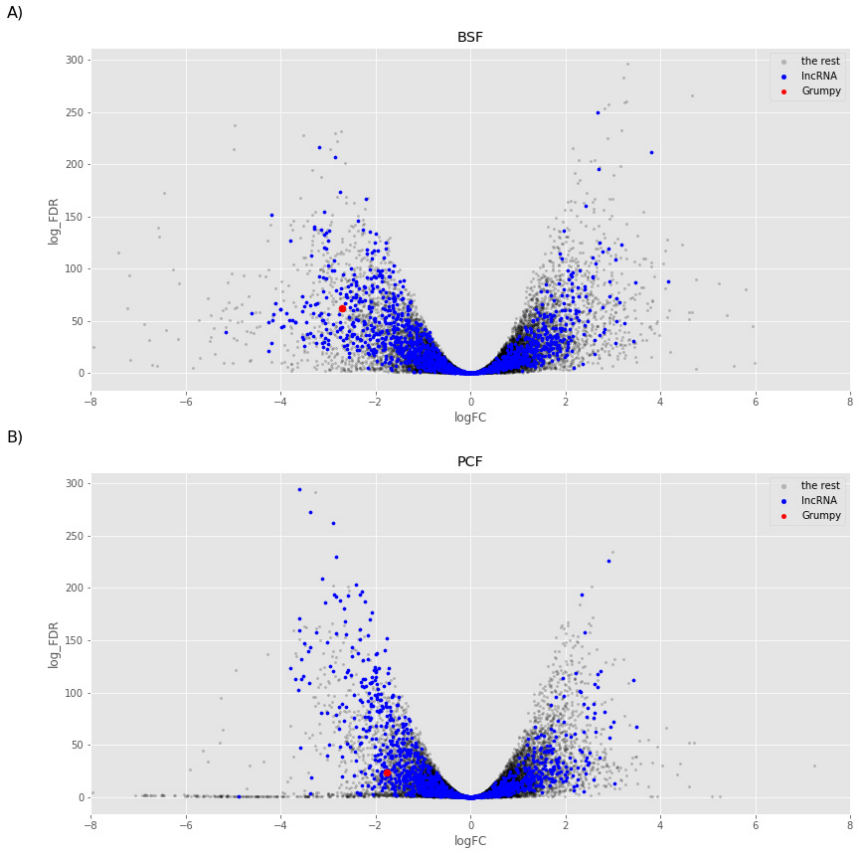
Sub-polysome abundance test. The volcano plots report the log2 fold change (logFC) on the x-axis and the minus log10 of the false discovery rate (log_FDR) on the y-axis obtained from the comparison of the sub-polysomal samples with the polysomal samples for Bloodstream From (BSF, **A**) and Bloodstream From (PCF, **B**) samples. Blue dots highlight the long non-coding RNAs (lncRNA), red dot highlights the *grumpy* gene described Guegan *et al*. ^31^, and grey dots highlight the rest of the genes in the sample.

**Figure 14.**
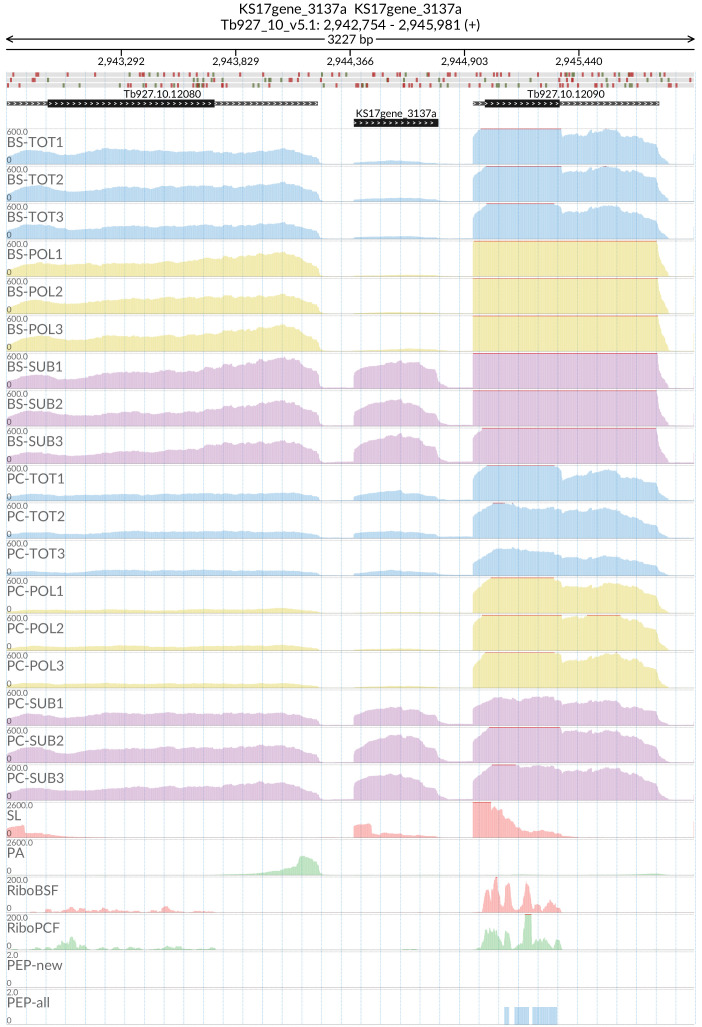
For the *grumpy* gene KS17gene_3137a the figure shows the genome coverage for the total (TOT), polysomal (POL), and subpolysomal (SUB) samples (biological replicates 1 to 3) of the bloodstream (BS) and procyclic (PC) form life stages. The figure also reports the genome coverage of the Splice Leader (SL) and poly(A) mRNA tails and/or poly(A) genomic tract (PA) containing reads assembled from the samples. Also shown are the ribosome profiling reads for the Bloodstream Form (RiboBSF) and Procyclic Form (RiboPCF) life stages as described in Vasquex *et al*. 2014. The last two genomic tracks report the peptide identifications for new predicted open reading frames (PEP-new) and for all the open reading frames (PEP-all) in TritrypDB. The maximum height of each of the gene tracks is reported on the top left of each track. The top of the figure shows an ideogram of the gene structures. The three grey genomic tracks at the top report ATG codons in green and stop codons in red.

**Figure 15.**
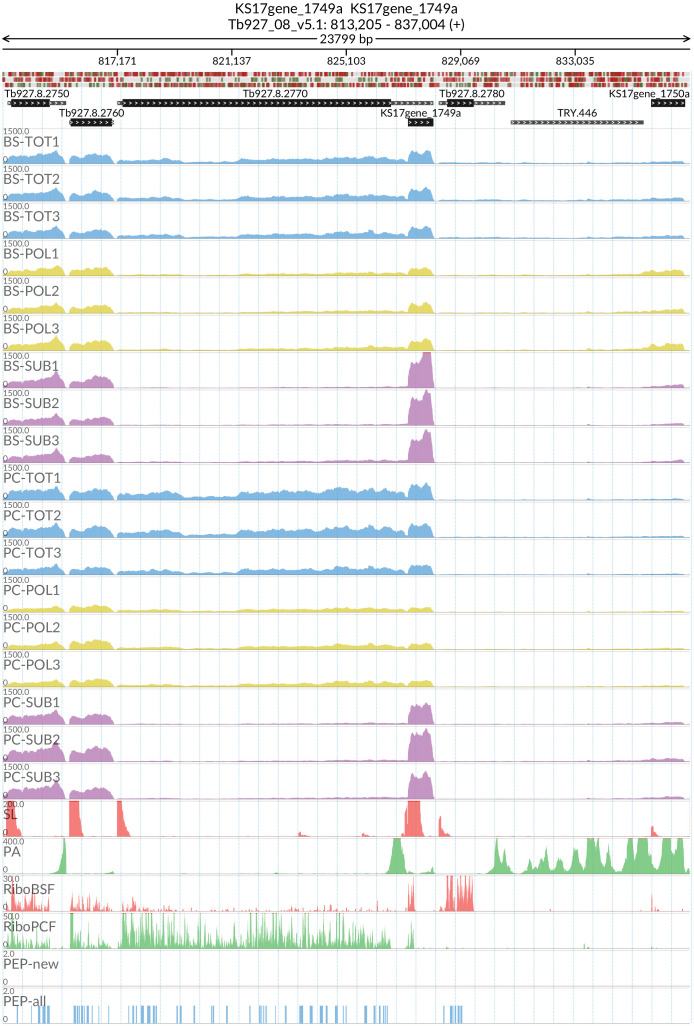
For the long non coding RNA KS17gene_1749a at the 5’ of the Tb927.8.2780 (RNA-binding protein RBP10) gene, the figure shows the genome coverage for the total (TOT), polysomal (POL), and subpolysomal (SUB) samples (biological replicates 1 to 3) of the bloodstream (BS) and procyclic (PC) form life stages. The figure also reports the genome coverage of the Splice Leader (SL) and poly(A) mRNA tails and/or poly(A) genomic tract (PA) containing reads assembled from the samples. Also shown are the ribosome profiling reads for the Bloodstream Form (RiboBSF) and Procyclic Form (RiboPCF) life stages as described in Vasquex *et al*. 2014. The last two genomic tracks report the peptide identifications for new predicted open reading frames (PEP-new) and for all the open reading frames (PEP-all) in TritrypDB. The maximum height of each of the gene tracks is reported on the top left of each track. The top of the figure shows an ideogram of the gene structures. The three grey genomic tracks at the top report ATG codons in green and stop codons in red.

**Figure 16.**
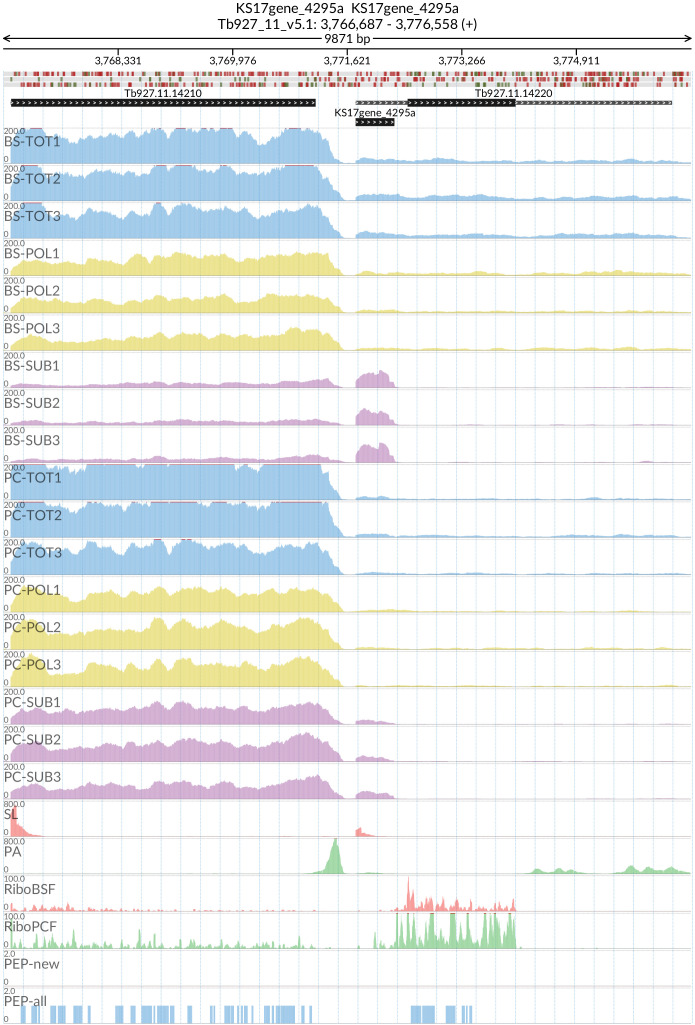
For the long non coding RNA KS17gene_4295a at the 5’ of the Tb927.11.14220 (REG9.1) gene, the figure shows the genome coverage for the total (TOT), polysomal (POL), and subpolysomal (SUB) samples (biological replicates 1 to 3) of the bloodstream (BS) and procyclic (PC) form life stages. The figure also reports the genome coverage of the Splice Leader (SL) and poly(A) mRNA tails and/or poly(A) genomic tract (PA) containing reads assembled from the samples. Also shown are the ribosome profiling reads for the Bloodstream Form (RiboBSF) and Procyclic Form (RiboPCF) life stages as described in Vasquex *et al*. 2014. The last two genomic tracks report the peptide identifications for new predicted open reading frames (PEP-new) and for all the open reading frames (PEP-all) in TritrypDB. The maximum height of each of the gene tracks is reported on the top left of each track. The top of the figure shows an ideogram of the gene structures. The three grey genomic tracks at the top report ATG codons in green and stop codons in red.

We then focused on the analysis of the transcripts enriched in the sub-polysomal samples. We created two models to test for differential abundance between the sub-polysomal and polysomal samples in the BSF ( *Extended data*: Table 4 ^60^) and PCF ( *Extended data*: Table 5 ^60^) life stages. As illustrated in Figure 13, several long non-coding genes are more abundant in the sub-polysomal samples with respect to the polysomal samples, including the *grumpy* transcript ( Figure 14) that sits at the 5’ end of RBP7A (Tb927.10.12080) and has been shown to be important for the progression from the slender form to the stumpy form of the parasite ^26^. The *grumpy* transcript made us wonder which other sub-polysome enriched transcripts might have a lncRNA at the 5’ end and be associated with this life stage transition. We identified two candidate genes: RBP10 (Tb927.8.2780) with the lncRNA KS17gene_1749a ( Figure 15) and REG9.1 (Tb927.11.14220) with the lncRNA KS17gene_4295a ( Figure 16), both of which have been previously associated with the transition between the BSF and PCF life stages ^61, 62^. We then asked which other genes, involved in RNA processing, have a 5’ lncRNA that is more abundant in the sub-polysome samples. To this aim, we created a new model to test for differential abundance between sub-polysomal and polysomal samples (mixed BSF and PCF). After extracting the lncRNAs that are more abundant in the sub-polysomal samples ( *grumpy*-like lncRNAs) we then reported the genes at their 3’ ends and describe these as “ *grumpy*-like genes”. Finally, we intersected these *grumpy*-like genes with a list of 322 potential post-transcriptional regulators in *T. brucei* ^63^ to identify 27 new proteins that may contribute to *T. brucei* life stage transitions ( *Extended data*: Table 6 ^60^).

### Identification of new protein coding genes

We were interested in evaluating whether there is proteomic evidence for the new hypothetical protein-coding genes identified in our dataset. To achieve this, we analyzed our protein half-life dataset ^64^ by running MaxQuant with a database of open reading frames (ORFs) for the TREU927 genome downloaded from TryTripDB. The genomic coordinates of the ORF peptides were then intersected with the genomic coordinates of the hypothetical new protein coding genes. Further, we filtered out unannotated genes in the main 11 chromosomes of *T. brucei*, without a splice leader site and without ribo-seq data. This analysis led to the identification of 11 new hypothetical protein coding genes reported in *Extended data*: Table 7 ^60^.

As examples, two of these hypothetical protein coding genes (TRY.375 and MSTRG.94) are described further.

### TRY.375

**Figure 17.**
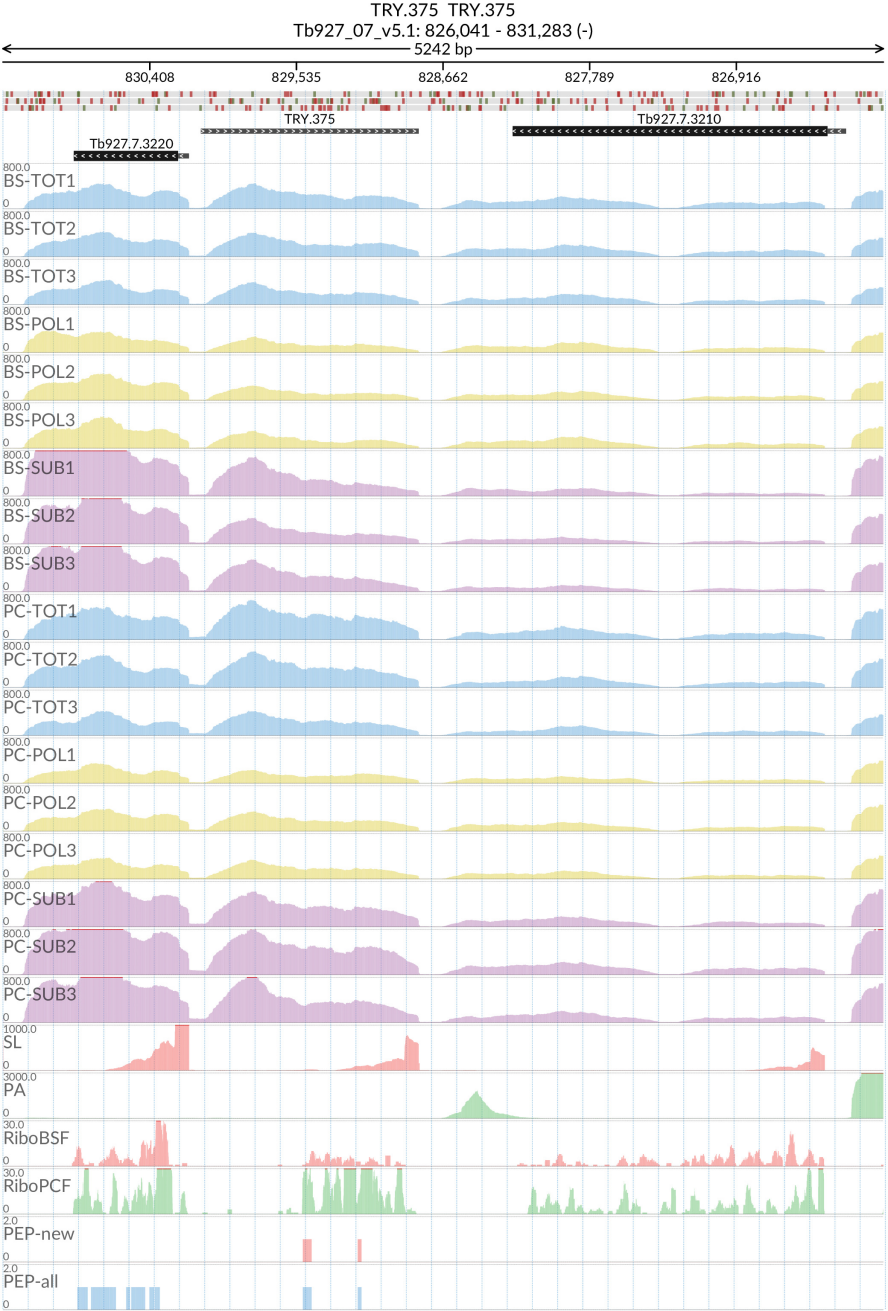
For the new predicted protein coding gene TRY.375, the figure shows the genome coverage for the total (TOT), polysomal (POL), and subpolysomal (SUB) samples (biological replicates 1 to 3) of the bloodstream (BS) and procyclic (PC) form life stages. The figure also reports the genome coverage of the Splice Leader (SL) and poly(A) mRNA tails and/or poly(A) genomic tract (PA) containing reads assembled from the samples. Also shown are the ribosome profiling reads for the Bloodstream Form (RiboBSF) and Procyclic Form (RiboPCF) life stages as described in Vasquex *et al*. 2014. The last two genomic tracks report the peptide identifications for new predicted open reading frames (PEP-new) and for all the open reading frames (PEP-all) in TritrypDB. The maximum height of each of the gene tracks is reported on the top left of each track. The top of the figure shows an ideogram of the gene structures. The three grey genomic tracks at the top report ATG codons in green and stop codons in red.

The start and end of the putative gene were designated at Tb927_07_v5.1:828803.. 830064 by Spliced Leader (SL)/Poly-A (PA) mapping. The putative TRY.375 gene ( Figure 17) contains a predicted open reading frame of 522 base pairs encoding for a protein of 173 amino acids (19.51 kDa). The TRY.375 protein product is predicted to have an uncleaved signal peptide and three transmembrane domains. Blastp analysis of the protein product returned low percentage identity (<50%) matches with genes in *T. grayi* (DQ04_00451000), *T. conorhini* (accession: XP_029230363.1) and *T. theileri* (TM35_000192250). Synteny analysis of the TRY.375 locus performed at TryTripDB revealed another gene (TevSTIB805.7.3380) in the *T. evansi* genome with 100% homology with the predicted TRY.375 gene product. Also, a tblastn search of the TRY.375 predicted gene identified 2 more hits with 100% homology in the genomes of *T. brucei* 427_2018, 427 (Tb427) and *T. brucei* g *ambiense* DAL972 (Tbg972), corresponding to unannotated regions in these genomes. We propose that TRY.375 is a novel transmembrane-protein coding gene present in *T. brucei* and *T. evansi.*

### MSTRG.94

**Figure 18.**
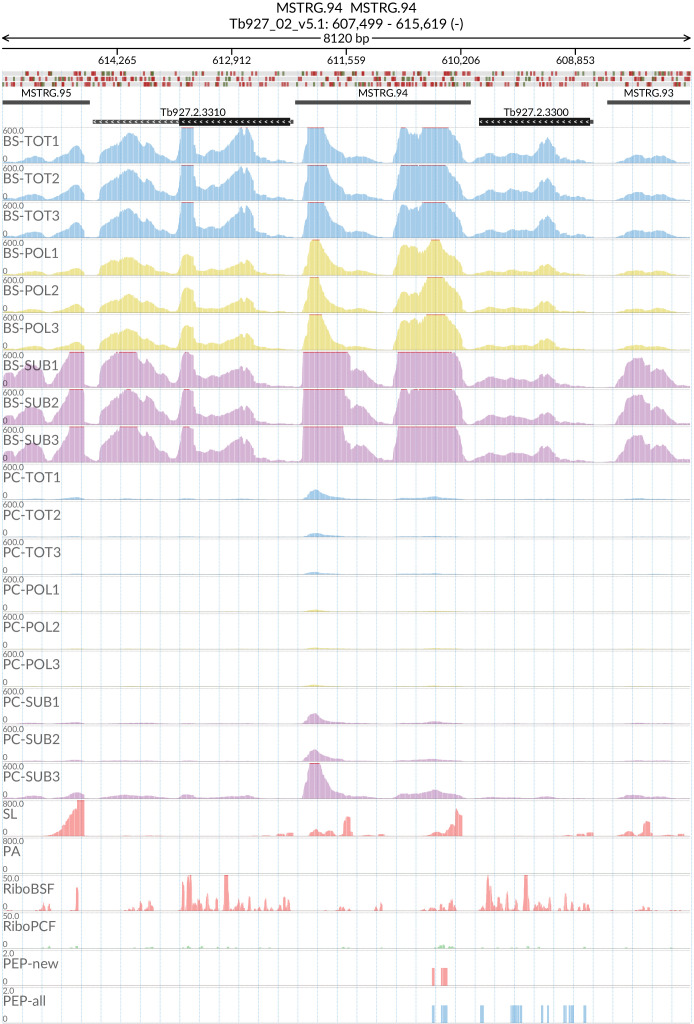
For the new predicted protein coding gene MSTRG.94, the figure shows the genome coverage for the total (TOT), polysomal (POL), and subpolysomal (SUB) samples (biological replicates 1 to 3) of the bloodstream (BS) and procyclic (PC) form life stages. The figure also reports the genome coverage of the Splice Leader (SL) and poly(A) mRNA tails and/or poly(A) genomic tract (PA) containing reads assembled from the samples. Also shown are the ribosome profiling reads for the Bloodstream Form (RiboBSF) and Procyclic Form (RiboPCF) life stages as described in Vasquex *et al*. 2014. The last two genomic tracks report the peptide identifications for new predicted open reading frames (PEP-new) and for all the open reading frames (PEP-all) in TritrypDB. The maximum height of each of the gene tracks is reported on the top left of each track. The top of the figure shows an ideogram of the gene structures. The three grey genomic tracks at the top report ATG codons in green and stop codons in red.

Peptides corresponding to potential new gene MSTRG.94 ( Figure 18) mapped with high confidence to 6 regions within the span Tb927_02_v5.1:592500..617500. Investigation of this section of chromosome 2 revealed it is highly repetitive and contains 6 copies of a 65kDa Invariant Surface Glycoprotein gene with a pairwise protein Identity computed by Clustal Omega between 73% and 99%. This suggests that what had previously been assumed to be untranslated intergenic regions of DNA may in fact encode for protein. SL and PA mapping allowed us to define 6 MSTRG.94 gene boundaries as described in *Extended data*: Table 7 ^60^. All of these putative gene regions were identical and we have designated them MSTRG.94_1 through MSTRG.94_6. The putative MSTRG.94 genes contain a predicted ORF of 378 base pairs encoding for a protein of 125 amino acids (14.17 kDa). The predicted protein does not contain any transmembrane domains or signal peptides. A tblastn search with the ORF sequence against trypanosome genomes revealed matching sequences in the genomes of Tb427 and *T. evansi*. As with Tb927, the sequences appear between copies of 65kDa Invariant Surface Glycoprotein genes in chromosome 2. In Tb427 the sequences are annotated as hypothetical proteins and in *T. evansi* as unspecified products, while in Tbg972 the regions are unnanotated.

## Discussion

In this paper we present RNA-seq data on the total, polysomal and sub-polysomal mRNA content of *T. brucei* bloodstream and procyclic form life stages. Comparison with similar experiments performed earlier by Antwi *et al.* ^12^ showed better experimental reproducibility between PCF life stage data (r ^2^=0.9) than BSF life stage data (r ^2^=0.7) ( Figure 5). A possible source of discrepancy may be different cell culture protocols for the BSF cells. Nevertheless, our dataset showed very good reproducibility ( Figure 2), and we were successful in identifying a pool of efficiently transcribed and spliced mRNAs. This is demonstrated by the virtual absence of reads covering the intron regions of the two experimentally validated intron containing genes ( Figure 3 and Figure 4) ^65^.

By using clustering and dimensionality reduction techniques ( Figure 8 and Figure 10), we were able to identify the sub-polysome samples as the most diverse in our dataset. In particular, we found the presence of several long non-coding mRNAs in the sub-polysomal fractions of both BSF and PCF samples ( *Extended data*: Table 3 ^60^). This class of mRNA has been overlooked in *T. brucei* until recently, and one particular long non-coding mRNA ( *grumpy*) has been shown to regulate the transformation from the slender to the stumpy life stage of the parasite ^31^. Interestingly, the RNA-binding protein RBP10 (Tb927.8.2780), that has been shown to bind mRNAs and promote their degradation, acts as a molecular switch whereby RBP10 expression in BSF causes differentiation to PCF, while the overexpression in PCF causes differentiation to BSF ^61^. While RBP10 itself was not found in our sub-polysome enriched transcript list, the lncRNA (KS17gene_1749a) which is predicted to be at the 5’ end of RBP10 may have a similar regulatory function as the *grumpy* lncRNA transcript. Therefore, we have assembled a list of other putative RNA regulatory proteins that may be of interest for those working on *T. brucei* life stage transitions ( *Extended data*: Table 6 ^60^).

It should be noted that several lncRNAs are located next to other annotated genes in the genome. We speculate that those lncRNAs might represent transcript isoforms of genes that are actively regulated; the lncRNAs themselves might be retained and/or removed from a transcript to promote/decrease transcript stability. The excised lncRNA might then be targeted for degradation, and this could be the reason why the lncRNAs are highly abundant in the sub-polysomal fraction. This hypothesis might also explain why the shortest half-life mRNAs are more abundant in the sub-polysome fractions. Once a lncRNA has been excised from the parent transcript, both the lncRNA and the parent transcript may be targeted for degradation. In any case, we anticipate that the study of lncRNAs transcripts that are more abundant in the sub-polysomal fraction may uncover new mechanisms of transcript stability and regulation in *T. brucei*. To facilitate such studies and to shortlist candidate genes for further experiments, we provided a table of protein coding genes involved in mRNA processing with the closest lncRNA that is more abundant in the sub-polysome fractions ( *Extended data*: Table 5 ^60^).

Another class of RNA we found to be enriched in the sub-polysomal fractions are snoRNAs. The presence of this class of RNA in the sub-polysomal fraction might also be explained by a degradation mechanism. For example, snoRNAs guide the peculiar trypanosome rRNA maturation events, facilitating the methylation and pseudouridylation modification of rRNA ^66, 67^. Because polyadenylation by snoRNA is a way of marking the RNA for degradation in yeast and humans ^68, 69^, it is possible that a similar mechanism acts in *T. brucei,* and that our poly-A enrichment step has captured this class of RNAs before they have been targeted to the exosome for degradation ^70^.

Finally, we hope that our dataset will be useful for the annotation of the *T. brucei* genome, as demonstrated by the identification of 30 new hypothetical protein-coding genes.

## Data availability

### Underlying data

All FASTQ files data are deposited at the NCBI SRA database ^71^ under the bioproject accession number PRJNA634997

Analysis pipeline, links to the raw data and code used to generate the paper figures are available at https://github.com/mtinti/polysome, reproducible using the mybinder badge in GitHub and archived in Zenodo.

Zenodo: mtinti/polysome: pre-submission. http://doi.org/10.5281/zenodo.4235160 ^72^

This project contains the following data:

- (B,P)_(pol, sub, tot)_(1,2,3)
  - counts.txt (The read counts for the genes)
  - counts_CDS.txt (The read counts for the gene coding sequences)
- Figures (The folder containing the figures of the paper)
- Figures_Paper_def.ipynb (The jupyter notebook producing the figures of the paper)
- InData
  - GC_content_927.txt (list of guanine-cytosine content values of the genes in T. brucei)
  - GS_gene_list.txt (list of the hypothetical long non-coding mRNAs in T. brucei according to Guegan F. *et al.* 2020)
  - PTR.txt (list of the genes with a predicted gene expression regulation effect in T. brucei according to Erben, E.D., *et al.* 2014)
  - PolisomeLiterature
    - BSF.csv (The supplementary Table 5 of Antwi *et al.* 2016 for the bloodstream life stage)
    - GeneByLocusTag_Summary.txt ( A mapping dictionary to update the gene ids in the supplementary Table 5 of Antwi *et al.* 2016)
    - PCF.csv (The supplementary Table 5 of Antwi *et al.* 2016 for the procyclic life stage)
  - Proteomics
    - peptides_bsf_trim.zip (peptide identification output of MaxQuant in the bloodstream life stage)
    - peptides_pcf_trim.zip (peptide identification output of MaxQuant in the procyclic life stage)
  - TriTrypDB-46_TbruceiTREU927.gff (generic feature format file downloaded from TriTrypDB)
  - TriTrypDB-46_TbruceiTREU927_GO.gaf (Gene Ontology file downloaded from TriTrypDB)
  - TriTrypDB-46_TbruceiTREU927_GO2.gaf (Gene Ontology file modified and used as input for GOATOOLS)
  - go-basic.obo (Ontology file downloaded from http://geneontology.org/docs/download-ontology/)
  - goterm_enrich.txt (list of enriched GO terms in the gene clusters)
  - mRNA_Half_Life
    - mRNAhl_lookup.txt ( A mapping dictionary to update the gene ids in the supplementary Table 5 of Antwi *et al.* 2016)
    - mrnaBSFhl.txt (list mRNAs half-lives for bloodstream form as reported in supplementary Table 5 of Antwi *et al.* 2016)
    - mrnaPCFhl.txt (list mRNAs half-lives for procyclic form as reported in supplementary Table 5 of Antwi *et al.* 2016)
  - ribo_counts_927.txt (Read counts for the re-analysis of the ribo-seq dataset)
- Tables (The folder containing the tables of the paper)
- environment.yml (The conda environment file that lists the packages to reproduce the analysis on mybinder)
- make_pipline2.py (python script to assemble the rna-seq analysis pipeline)
- multiQC.ipynb (The jupyter notebook that runs the quality control )
- multiqc_config.yaml (The multiQC configuration file )
- multiqc_fastqc.yaml (The multiQC configuration file for the fastqc package )
- mylib
  - extract_barcodes_def2.py (The python script to extract the RNA-seq reads containing the splice leader sequences or the poly-A tracts)
- polysome_mqc (folder containg the multiQC output files)
- package_versions.txt (a text file listing all the versions of the software used for the analysis)
- postBuild (configuration files for mybinder)
- tb927_3_ks_st_sc_st_tr.gtf (Gene Transfer annotation file of T. brucei listing the new transcribed regions identified in this work)
- tb927_5.fa (Genomic sequences of T. brucei downloaded from TriTrypDB)
- tb927_5.fa.fai (index file Genomic sequences of T. brucei)
- tb927_5.gtf (Gene Transfer annotation file of T. brucei downloaded from TriTrypDB)
- templates
  - scallop.sh (the bash script to run scallop for the identification of new transcribed regions))
  - template_rnaseq.sh (the bash script to run the RNA-seq analysis pipeline)
  - trinity_template.sh (the bash script to run trinity for the identification of new transcribed regions)
- README.md (the github readme file)
- utilities.py (python script with helper functions for the data analysis )
- vars5.txt (list the input parameters for the make_pipline2.py file)
- wcar.png (Wellcome Centre for Anti-Infectives Research logo)

The code and the data used to generate the paper figures that visualise the RNA-seq coverage are available at https://github.com/mtinti/polysome_coverage, https://github.com/mtinti/polysome, reproducible using the mybinder badge in github and archived in zenodo.

Zenodo: mtinti/polysome_coverage: pre-submission. http://doi.org/10.5281/zenodo.4428343 ^73^

This project contains the following data:

- (B/P)_(pol/sub/tot)_(1/2/3)_sorted_pc_bg.bed (bed graph file for the coverage of the RNA-seq samples)
- Figures_Paper_Coverage.ipynb (The jupyter notebook that produce the coverage images )
- README.md (the GitHub readme file)
- Tb927.8.1510_paper_figures.png (coverage image for the Tb927.8.1510 gene)
- all_927_F_plus_R_SL.bed (bed graph file format for the coverage of the reads containing the spliced-leader sequences)
- all_F_plus_R_PoliA.bed (bed graph file format for the coverage of the reads containing the poli-A tract)
- all_pepe.bed (bed graph file format for the coverage of the peptides identified with mass spectrometry)
- environment.yml (The conda environment file that lists the packages to reproduce the coverage analysis on mybinder)
- new_genes.bed (bed graph file format for the coverage of the peptides identified with mass spectrometry for new predicted protein coding gene)
- package_versions.txt (a text file listing all the versions of the software used for the analysis)
- riboBSF_927.bed (bed graph file format for the coverage of ribo-seq samples in the bloodstream samples)
- riboPCF_927.bed (bed graph file format for the coverage of ribo-seq samples in the procyclic sample)
- svist4getConf (configuration folder for the svist4get package)
- tb927_3.gff (Gene Transfer annotation file of T. brucei downloaded from TriTrypDB)
- tb927_5.fa (Genomic sequences of *T. brucei* downloaded from TriTrypDB)
- tb927_5.fa.fai (index file Genomic sequences of *T. brucei* downloaded from TriTrypDB)
- tb927_5.gtf (Gene Transfer annotation file of *T. brucei* downloaded from TriTrypDB and supplemented with the new discovered expressed sequences)
- util.py (python script with helper functions for the gene coverage analysis )

wcar.png (Wellcome Centre for Anti-Infectives Research logo)

The QC output is avaiable at github https://github.com/mtinti/polysome_qc, visualizable at https://polysome-qc.onrender.com and archived in zenodo.

Zenodo: mtinti/mtinti-polysome_qc. https://doi.org/10.5281/zenodo.4235212 ^74^

This project contains the following data:

- report.html (the home page of the visualization report)
- report_data (the configuration folder congaing the report data)

Licence: MIT.

### Extended data

Zenodo: mtinti/ polysome_extended: v0.1. https://doi.org/10.5281/zenodo.4526137 ^60^

This project contains the following extended data:

- **Table 3. Cluster analysis.** Data used for the cluster analysis. The first column reports the gene identification number and 18 columns with the normalized values for the biological replicates in the format of [B/P]_[tot/pol/sub]_[1/2/3] were B: bloodstream form, P: procyclic form, tot: total RNA sample, pol: polysomal sample, sub: subpolysomal sample, 1,2,3: biological replicate identifiers. The table also reports the predicted cluster identification number (label), a binary column reporting whether the gene is identified or not in the (is_ks), the gene description (desc), a binary column reporting whether the gene is annotated as an H/ACA-like snoRNA, a binary column reporting whether the gene is annotated as a snoRNA and a binary column reporting whether the gene is annotated as non-coding (Noncoding) RNA.
- **Table 4. Polysome/sub-polysome transcript differential abundance in BSF cells.** Comparison between the polysome and sub-polysome samples in the bloodstream form life stage: logFC, the log fold-change for each gene in the two groups being compared. logCPM, the log-average abundance for each gene in the two groups being compared. LR, likelihood ratio statistic. PValue, exact p-value for differential expression test. FDR, the p-value adjusted for multiple testing with the Benjamini–Hochberg method (false discovery rate).
- **Table 5. Polysome / Sub-polysome Transcript Differential Abundance in PCF.** Comparison between the polysome and subpolysome samples in the procyclic form life stage the: logFC, the log-abundance ratio, i.e. fold change, for each gene in the two groups being compared; logCPM, the log-average concentration/abundance for each gene in the two groups being compared; LR, likelihood ratio statistics; PValue, exact p-value for differential expression test; FDR, the p-value adjusted for multiple testing with the Benjamini–Hochberg method.
- **Table 6. Grumpy-Like lncRNA Genes.** Comparison between the polysome and sub-polysome samples for the ‘ *grumpy*-like’ lncRNAs (gene_ks) reporting the: logFC, the log fold-change for each gene in the two groups being compared. logCPM, the log-average abundance for each gene in the two groups being compared. LR, likelihood ratio statistic. PValue, exact p-value for differential expression test. FDR, the p-value adjusted for multiple testing with the Benjamini–Hochberg method. The table also shows the predicted affected genes (gene_sensitive ) at the 3’ of the ‘ *grumpy*-like’ genes and the description (desc) of the affected gene and the regulation type (reg_type) reported in 63 for potential post-transcriptional regulators in *T. brucei.*
- **Table 7. New Protein Coding Genes.** The ID of the new predicted protein coding genes (Gene), the number of peptides identified in mass spectrometry (Peptides found by MS), the genomic coordinates (Coordinates), the gene length in base pairs (Gene length), the open reding frame orientation (Orient), the coding sequence coordinate (CDS coordinates), the open reading frame length in base pairs (ORF length), the predicted protein length in amino acid residues (Predicted protein length), the predicted protein molecular weight in Kilodalton (Predicted protein estimated weight), the identification number of other genes with high homology with the predicted gene (Similar genes), the number of transmembrane domain predicted with the Phobius algorithm (Phobius predictions), the signal peptide prediction results computed with the SignalP 3 algorithm (SignalP 3.0 predictions) or SignalP 5 algorithm (SignalP 5.0 predictions), are reported for the new predicted protein coding gene manually curated.

Data are available under the terms of the Creative Commons Zero "No rights reserved" data waiver (CC0 1.0 Public domain dedication).
