# Supplementary material for: Polysomal mRNA Association and Gene Expression in Trypanosoma brucei
Source: Wellcome Open Res. 2022 Feb 1;6:36. Originally published 2021 Feb 22. [Version 3] doi: 10.12688/wellcomeopenres.16430.3 (PMC8240603; doi:10.12688/wellcomeopenres.16430.3)
Supplement: Peer review history

# Peer review: Reviewer response for version 3

**DOI:** 10.21956/wellcomeopenres.19517.r48406

**Published:** 2022-02-15

**Authors:** Osvaldo P de Melo Neto, Antonio Rezende

**Affiliations:** 1 Department of Microbiology, Instituto Aggeu Magalhães, Fiocruz, Recife, PE, 50740-465, Brazil; 2 Department of Microbiology, Instituto Aggeu Magalhães, Fiocruz, Recife, Brazil

I have seen the revised version of the manuscript and the replies to the issues and comments raised by me and my colleague Antonio Rezende. I can confirm that I am satisfied with the answers and modifications carried out by the authors in the revised version and that I can fully "Approve" the manuscript without reservations.

Is the work clearly and accurately presented and does it cite the current literature?

Yes

If applicable, is the statistical analysis and its interpretation appropriate?

Yes

Are all the source data underlying the results available to ensure full reproducibility?

Yes

Is the study design appropriate and is the work technically sound?

Yes

Are the conclusions drawn adequately supported by the results?

Partly

Are sufficient details of methods and analysis provided to allow replication by others?

Partly

Reviewer Expertise:

Molecular Biology, Protozoology, Bioinformatics

We confirm that we have read this submission and believe that we have an appropriate level of expertise to confirm that it is of an acceptable scientific standard.

# Peer review: Reviewer response for version 3

**DOI:** 10.21956/wellcomeopenres.19517.r48404

**Published:** 2022-02-08

**Authors:** Esteban Erben

**Affiliations:** 1 IIBIO-UNSAM, Buenos Aires, Argentina

The authors have satisfactorily addressed most of my concerns.

Is the work clearly and accurately presented and does it cite the current literature?

Partly

If applicable, is the statistical analysis and its interpretation appropriate?

Yes

Are all the source data underlying the results available to ensure full reproducibility?

Yes

Is the study design appropriate and is the work technically sound?

Yes

Are the conclusions drawn adequately supported by the results?

Partly

Are sufficient details of methods and analysis provided to allow replication by others?

Yes

Reviewer Expertise:

Molecular Parasitology

I confirm that I have read this submission and believe that I have an appropriate level of expertise to confirm that it is of an acceptable scientific standard.

# Peer review: Reviewer response for version 1

**DOI:** 10.21956/wellcomeopenres.18079.r44178

**Published:** 2021-07-05

**Authors:** Osvaldo P de Melo Neto, Antonio Rezende

**Affiliations:** 1 Department of Microbiology, Instituto Aggeu Magalhães, Fiocruz, Recife, PE, 50740-465, Brazil; 2 Department of Microbiology, Instituto Aggeu Magalhães, Fiocruz, Recife, Brazil

The manuscript by Tinti *et al. *sought to generate profiles of mRNAs associated with polysomal and non-polysomal fractions from the parasitic protozoa “Trypanosoma brucei *”*; polysomal mRNAs indicating those being actively translated and used for protein synthesis. The study compares the profiles seen for the two major life forms of “T. brucei” (bloodstream and procyclic) using as biological material fractions of cycloheximide-treated cells. Pooled samples consisting of polysomal and non-polysomal fractions, separated by sucrose gradient, were submitted to RNA extraction, cDNA synthesis and next generation sequencing in order to qualitatively and quantitatively define mRNAs (or polyadenylated RNAs) bound or not bound to the polysomes. In all, the authors investigated three different samples for each parasite life form: total polyA+ mRNAs, sub-polysomal mRNAs and polysomal mRNAs. A large range of bioinformatic tools were used to analyze the results and these were also compared with previous published data.

      The data generated by the reported research, with the comparison between polysomal and sub-polysomal mRNAs from the two different life forms, expands on previous works assessing polysome bound mRNAs in *T. brucei*, increases the number of experimentally validated and mapped mRNAs and identifies and investigates non-coding mRNAs in the sub-polysomal fraction, with possible roles in regulation of specific messages. It constitutes an important tool for a large number of investigators working on mRNA translation and gene expression regulation in these and related protozoa. The approach is especially relevant considering that these are eukaryotes with very peculiar mechanisms for regulation of gene expression, with previous evidence highlighting a strong role for this regulation during mRNA translation, therefore being model organisms for mechanisms targeting regulation of translation. Nevertheless, for publication, the manuscript needs improvements regarding several issues, as detailed below.

**Abstract:**           

             The abstract should be thoroughly revised with an inclusion of more relevant results. As detailed further below, a focus only on the presence of long non-coding RNA in the sub-polysomal fraction (stated twice) is misleading since these should be mainly found in this fraction to begin with. More relevant results include: the proper identification/confirmation of long non-coding, polyadenylated RNAs (with the 5’ SL), with substantial number of reads and their mapping, highlighting possible roles regarding the regulation of specific protein coding genes (it is not clear by the text to which extent novel lncRNAs were identified, but if so this has to be highlighted as well as the association reported between lcnRNAs and neighboring protein coding transcripts); the substantial increase of genome loci found to be transcribed, as reported in the main text; and the identification of novel hypothetical proteins.

**Methods:**

        The approach applied in the manuscript was based on several bioinformatic tools and computational steps. Some of the tools used are quite old, such as GMAP, but more relevant was the use of several computational tools and analyses without a proper justification for their use. For example, why did the authors decide to use two different *de novo* assemblers (Trinity and Scallop) for the transcriptomic data? A consensus between the transcript predictions from both tools and including prior predictions was then expected, but it is not clear if this is what the authors did and should be clarified. At the end of the “de novo” approach, a new genome annotation file was generated, but it is not clear if it was used for the final read counts using the “featureCounts” tool and this also needs clarification. Regarding the description of the cDNA library preparation, important details such as read length and which type of library was employed cannot be found in the methods section. Base on the text it is understood that the libraries used were *paired-end*, but then for quality control, the authors mentioned they used RPKM as normalization step, which is applicable to *single-end* libraries. These details need to be better clarified.

      An important point of concern is the use of two different strains, one for each of the two *T. brucei* life forms. No considerations or comments are made on how this can impact on the differences found between the same fractions from the two different life forms investigated. To what extent could the differences seen between the two life forms, and related to the results from figure 5, could be associated with the use of different strains?

**Results:**

      The very large number of figures can be a distraction and keep the reader from focusing on what is important. The authors should consider reducing those. Some suggestions: Figures 1 is not necessary; for Figures 6 and 7, only one representative figure could be kept, or both could be removed altogether; Figure 9 also does not need to be shown.  

      For validation purposes, wouldn´t it be relevant mentioning or showing the profile of known, stage specific genes, which would preferentially be present in the polysomal fractions of either bloodstream or procyclic forms? For instance, known surface antigens?

      The authors used as examples on mapping quality control, two *T. brucei* genes known to have introns. However, it is not clear why one should see substantial read coverage for an intron in sub-polysomal and total RNA samples. It was understood that the work aimed to compare sub-polysomal or polysomal fractions of presumably cytoplasmic and mature mRNAs, selected through their poly-A tails. So, very low or no intron mapping would be expected overall and not only for the polysomal fractions. Wouldn’t the presence of introns indicate a substantial amount of precursor or maybe nuclear mRNAs within the sub-polysomal fractions that need to be considered somehow?

     A relevant observation from the results shown in Figure 4 is the overlap in the UTRs from the Tb927.8.1500 and Tb927.8.1510. How unique is this? Likewise, in Figure 3, for the Tb927.3.3160 gene, its intron might be associated with poly-A tracts. Again, what does this mean and is it seen elsewhere? These issues should be considered in the text.

     The authors mention an enrichment of lncRNAs in cluster 2, composed mainly by sub-polysomal mRNAs from bloodstream and procyclic forms. Biologically speaking, isn’t that to be expected, as no ribosomes should be attached to non-translatable lncRNAs? The authors propose a mechanism where a lncRNA would be part of a transcript, and its presence or absence could define different transcript isoforms. But which isoform would be translated, with or without the lncRNA segment? In this case would the lncRNA segment be considered a long non-coding RNA? Why would the lncRNA have a Spliced Leader and poly-A, as indicated by the results? These issues were discussed only superficially in the Discussion.

     The authors also proposes that the location of the segments encoding several lncRNA might indicate a role in regulation of neighboring genes. What is the basis for this? Has it been shown elsewhere or in Tryps? It seems quite speculative and not much related to this is seen in the discussion.

     Finally, it would be nice to have some data regarding the abundance of different functional classes of mRNA coding proteins when forms and fractions were compared, however, there is nothing on that in the discussion.

**Discussion:**

     Regarding the discussion, the work has several interesting results, but few of them are discussed sufficiently. For instance, the results mention a 34% increment in the number of transcribed loci, but there is no consideration regarding this very relevant results in the discussion, and no details can be found regarding function and curation of these loci.

**Minor Points:**

1. What is the difference between PCA and MDS? Do the axes have weights? This can change the interpretation of the result. Why didn’t authors use PCA?
2. In the methods, in the sentence “Before computing the fraction of transcripts in polysomes, the polysome read counts were divided by 0.7 and the sub-polysome read counts were divided by 0.3 …”, shouldn’t the counts be multiplied by 0.7 or 0.3, instead of divided by?
3. The following sentence is not understandable and needs to be clarified: "To identify the Grumpy Like genes we created a third model to study the differential transcript abundance between the sub-polysomal samples (mixed model of BSF and PCF) against the subpolysomal samples (mixed model of BSF and PCF)" present at "Sub-polysome / polysome differential abundance analysis and Grumpy Like GenesSub-polysome / polysome differential abundance analysis and Grumpy Like Genes" topic.
4. The first sentences of the Results section explaining in detail the use and properties of cycloheximide are not necessary and can be shortened since the cycloheximide use for polysomal gradients is common.
5. The statement “The poly(A) genomic tracts are often present in intergenic regions and can help to determine the 3’ gene boundaries” needs a reference.
6. In various Figure legends the citation of Vasquex (it is Vasquez) *et al. *2014 should include the reference number from the reference list.
7. Figure 8 - put the legend of the figure in the same orders of the sample names in the graph.
8. For Figure 10, review the order that the results are shown. It makes no sense having the two different sub-polysomal samples followed by the polysomal and total samples from procyclics and then polysomal and total samples from bloodstream. Either you alternate between each of the procyclic and bloodstream samples or show all procyclic and then all bloodstream samples.
9. The following sentence needs to be modified “The grumpy transcript made us wonder which other sub-polysome enriched transcripts might have a lncRNA at the 5’ end….”. Based on the “Grumpy” example, it refers to polysome enriched transcripts which have a sub-polysome enriched lncRNA at their 5’ end.
10. Figure 13 - The legend is confusing.
11. In the sentence "Synteny analysis of the TRY.375 locus performed at TryTripDB revealed another gene (TevSTIB805.7.3380) in the T. evansi genome with 100% homology with the predicted TRY.375 gene product.", the homology concept is misused as it is a binary concept bringing the idea of evolutionary relationship, so you cannot have degree of homology between two loci of different species. Either they are homologs or re not. The degree of similarity or identity should be informed. 
12. The novel MSTRG.94 gene and neighboring sequences seem to be transcribed only in Bloodstream cells and this should be highlighted.
13. In the Discussion, the statement “This is demonstrated by the virtual absence of reads covering the intron regions of the two experimentally validated intron containing genes” is not valid for the sub-polysomal transcripts and should be revised.

Is the work clearly and accurately presented and does it cite the current literature?

Yes

If applicable, is the statistical analysis and its interpretation appropriate?

Yes

Are all the source data underlying the results available to ensure full reproducibility?

Yes

Is the study design appropriate and is the work technically sound?

Yes

Are the conclusions drawn adequately supported by the results?

Partly

Are sufficient details of methods and analysis provided to allow replication by others?

Partly

Reviewer Expertise:

Molecular Biology of Prozooans; Gene expression regulation

We confirm that we have read this submission and believe that we have an appropriate level of expertise to confirm that it is of an acceptable scientific standard, however we have significant reservations, as outlined above.

# Response

**Published:** 2022-01-21

**Authors:** Michele Tinti

**Affiliations:** Dundee University, UK

1. The very large number of figures can be a distraction and keep the reader from focusing on what is important. 

We agree that we have many figures and most of them would be more appropriate as supplementary figures in other journals. However, as the WOR publication allows “as many figure as needed”, we decided to include a visual support for many of our statements.

  

1. For example, why did the authors decide to use two different de novo assemblers (Trinity and Scallop) for the transcriptomic data?

We found Trinity and Scallop identify different sets of transcribed regions. However, both assemblers were developed with eukaryotic genes with introns and struggled to correctly identify new genes in *T. brucei*. Particularly Trinity was prone to assemble transcripts encompassing several genes. For this reason, we run Scallop first to annotate new transcripts in regions without any previous annotation. Then, we repeated the same analysis with Trinity, again considering only regions without previous annotation. We have added an explanation to the paper materials and methods.

  

1. The authors mentioned they used RPKM as normalization step.

This was a mistake; it should have read as FPKM, and this has been corrected.

  

1. An important point of concern is the use of two different strains, one for each of the two T. brucei life forms. No considerations or comments are made on how this can impact on the differences found between the same fractions from the two different life forms investigated. To what extent could the differences seen between the two life forms, and related to the results from figure 5, could be associated with the use of different strains?

The data we produced form procyclic and bloodstream forms both come from the Lister 427 strain. This is now described more explicitly in Material and Methods.

  

1. For validation purposes, wouldn´t it be relevant mentioning or showing the profile of known, stage specific genes, which would preferentially be present in the polysomal fractions of either bloodstream

We did check a few key proteins that are bloodstream or procyclic life stage specific. Also, In Fig 5, we are already showing that our data is in good agreement with previous results (this has been also revisited as suggested by the reviewer in the minor point n 2).

  

1. However, it is not clear why one should see substantial read coverage for an intron in sub-polysomal and total RNA samples. It was understood that the work aimed to compare sub-polysomal or polysomal fractions of presumably cytoplasmic and mature mRNAs, selected through their poly-A tails. 

We believe that the sub-polysomal and total RNA fractions contain transcripts that for some reasons failed the splicing event and are targeted for degradation. We would expect these failed splicing events to be virtually absent from the actively transcribing polysomal fraction.

  

1. A relevant observation from the results shown in Figure 4 is the overlap in the UTRs from the Tb927.8.1500 and Tb927.8.1510. How unique is this? Likewise, in Figure 3, for the Tb927.3.3160 gene, its intron might be associated with poly-A tracts. Again, what does this mean and is it seen elsewhere? These issues should be considered in the text.

We address the difficulties to determine the 3’ UTRs end genomic coordinates in the new version of the article. We also reanalysed our data to consider only the lncRNAs and the CDS of protein coding genes for our statistical analysis. (see new chapter: Expression Analysis of lncRNAs and surrounding genes, Figure 17 and associated text).

  

1. The authors mention an enrichment of lncRNAs in cluster 2, composed mainly by sub-polysomal mRNAs from bloodstream and procyclic forms. Biologically speaking, isn’t that to be expected, as no ribosomes should be attached to non-translatable lncRNAs?

We have revised the discussion text highlighting a possible role for lncRNAs more abundant in the polysomal fraction.

  

1. The authors propose a mechanism where a lncRNA would be part of a transcript, and its presence or absence could define different transcript isoforms. But which isoform would be translated, with or without the lncRNA segment? In this case would the lncRNA segment be considered a long non-coding RNA? Why would the lncRNA have a Spliced Leader and poly-A, as indicated by the results? These issues were discussed only superficially in the Discussion.

We agree with the reviewer that no experimental evidence supports this speculative hypothesis and we have modified this part of the discussion.

  

1. The authors also proposes that the location of the segments encoding several lncRNA might indicate a role in regulation of neighboring genes. What is the basis for this? Has it been shown elsewhere or in Tryps? It seems quite speculative and not much related to this is seen in the discussion.

We will add a reference to “Statello et al. 2021, Nature Reviews Molecular Cell Biology. Gene regulation by long non-coding RNAs and its biological functions” providing pieces of evidence that  “Several lncRNAs control the expression of nearby genes by affecting their transcription”. We also provided more robust statistical analysis to correlate the expression of the lncRNAs and the genes at their 5’ or 3’

  

1. Regarding the discussion, the work has several interesting results, but few of them are discussed sufficiently. For instance, the results mention a 34% increment in the number of transcribed loci, but there is no consideration regarding this very relevant results in the discussion, and no details can be found regarding function and curation of these loci.

We have clarified that the majority of those loci represent miss-annotated UTRs. This is the reason why we used an unbiased proteomic approach to shortlist putative new protein-coding genes. It would be beyond our capacity to manually curate all the identified new loci and to better annotate the 3’ UTRs of many genes.

**Minor Points**

  

1. What is the difference between PCA and MDS? Do the axes have weights? This can change the interpretation of the result. Why didn’t authors use PCA?

We prefer the MDS analysis as the axes have a direct correlation with the differential expression of the considered genes, see https://www.biostars.org/p/287415/.

  

1. In the methods, in the sentence “Before computing the fraction of transcripts in polysomes, the polysome read counts were divided by 0.7 and the sub-polysome read counts were divided by 0.3 …”, shouldn’t the counts be multiplied by 0.7 or 0.3, instead of divided by?

The reviewer is correct, and this improves the correlation between our dataset and the dataset of Antwi et al. showed in Figure 5

  

1. The following sentence is not understandable and needs to be clarified: "To identify the Grumpy Like genes we created a third model to study the differential transcript abundance between the sub-polysomal samples (mixed model of BSF and PCF) against the subpolysomal samples (mixed model of BSF and PCF)" present at "Sub-polysome / polysome differential abundance analysis and Grumpy Like GenesSub-polysome / polysome differential abundance analysis and Grumpy Like Genes" topic.

We apologise for the poor construction and have clarified in the revised version (see new chapter: Expression Analysis of lncRNAs and surrounding genes, Figure 17 and associated text). [MF(5] [MT(6] 

  

1. The first sentences of the Results section explaining in detail the use and properties of cycloheximide are not necessary and can be shortened since the cycloheximide use for polysomal gradients is common.

We prefer to keep this sentence for people not familiar with this technique.

  

1. The statement “The poly(A) genomic tracts are often present in intergenic regions and can help to determine the 3’ gene boundaries” needs a reference.

We have added “ Radío et al. 2018 UTRme: A Scoring-Based Tool to Annotate Untranslated Regions in Trypanosomatid Genomes” to this sentence.

  

1. In various Figure legends the citation of Vasquex (it is Vasquez) et al. 2014 should include the reference number from the reference list.

We have fixed this.

  

1. Figure 8 - put the legend of the figure in the same orders of the sample names in the graph.

We have reordered the legend.

  

1. For Figure 10, review the order that the results are shown. It makes no sense having the two different sub-polysomal samples followed by the polysomal and total samples from procyclics and then polysomal and total samples from bloodstream. Either you alternate between each of the procyclic and bloodstream samples or show all procyclic and then all bloodstream samples.

This is the order determined by the column clustering, that we would rather keep as it is. We acknowledge that we did not describe the clustering of the columns that is now added to the materials and methods.

  

1. The following sentence needs to be modified “The grumpy transcript made us wonder which other sub-polysome enriched transcripts might have a lncRNA at the 5’ end….”. Based on the “Grumpy” example, it refers to polysome enriched transcripts which have a sub-polysome enriched lncRNA at their 5’ end.

We have rephrased this sentence.

  

1. Figure 13 - The legend is confusing.

We don’t find it confusing, we cordially ask the reviewer to specify better where the confusion arises.

  

1.  In the sentence "Synteny analysis of the TRY.375 locus performed at TryTripDB revealed another gene (TevSTIB805.7.3380) in the T. evansi genome with 100% homology with the predicted TRY.375 gene product.", the homology concept is misused as it is a binary concept bringing the idea of evolutionary relationship, so you cannot have degree of homology between two loci of different species. Either they are homologs or re not. The degree of similarity or identity should be informed. 

We now report on the degree of similarity.

  

1. The novel MSTRG.94 gene and neighboring sequences seem to be transcribed only in Bloodstream cells and this should be highlighted.

We have highlighted this.

  

1. In the Discussion, the statement “This is demonstrated by the virtual absence of reads covering the intron regions of the two experimentally validated intron containing genes” is not valid for the sub-polysomal transcripts and should be revised.

We have rephrased this sentence, referring only to the polysomal samples.

# Peer review: Reviewer response for version 1

**DOI:** 10.21956/wellcomeopenres.18079.r44183

**Published:** 2021-06-28

**Authors:** Magdalena Radwanska

**Affiliations:** 1 Biomedical Research Center (BMRC), Ghent University Global Campus, Incheon, South Korea; 2 Ghent University, Ghent, Belgium

This is a high-quality manuscript with sound methodology. The manuscript contributes to the understanding of mechanisms involved in regulation of a gene expression in *T. brucei* using cultured bloodstream forms and procyclic forms. Conducted bioinformatic analysis of RNS-seq on total, sub-polysomal and polysomal mRNA samples led to identification of several long non-coding RNAs (lncRNAs) and snoRNAs implicated in regulation of differentiation in *T. brucei*. In addition presented work includes also identification of 30 new hypothetical protein-coding genes facilitating further genomic annotation of *T. brucei.*

 In the host, *T. brucei* as an extracellular parasite surviving in direct contact with the immune system being confronted continuously with the host derived molecules. The latter were shown to have various impacts on parasite survival and differentiation. Hence, the parasite and host interplay and sensing of the environment play an important part in establishing of the infection. In order to make this manuscript more appealing to the wider public, it would be beneficial that authors discuss whether similar regulatory mechanisms involved in gene expression, are expected to operate during real in-host infection. Can observed *in vitro* regulatory mechanisms be extrapolated to the once existing during infection in the context of parasite differentiation, quorum sensing and overall survival?

Is the work clearly and accurately presented and does it cite the current literature?

Partly

If applicable, is the statistical analysis and its interpretation appropriate?

Yes

Are all the source data underlying the results available to ensure full reproducibility?

Yes

Is the study design appropriate and is the work technically sound?

Yes

Are the conclusions drawn adequately supported by the results?

Partly

Are sufficient details of methods and analysis provided to allow replication by others?

Yes

Reviewer Expertise:

Molecular Biology and Immunoparasitology

I confirm that I have read this submission and believe that I have an appropriate level of expertise to confirm that it is of an acceptable scientific standard.

# Peer review: Reviewer response for version 1

**DOI:** 10.21956/wellcomeopenres.18079.r44184

**Published:** 2021-06-11

**Authors:** Esteban Erben

**Affiliations:** 1 IIBIO-UNSAM, Buenos Aires, Argentina

In Tinti and co-workers, the authors look for novel coding sequences and potential lncRNAs by sequencing total RNA and different polysomal fractions from both PCF and BSF-trypanosome forms. The authors found that the grumpy lncRNA (the only lncRNA from *T. brucei *partially characterised so far potentially involved in the regulation of the slender-to-stumpy differentiation) is enriched in subpolysomal fractions in both life forms. They then looked for new lncRNAs displaying similar polysomal distribution finding a bunch of them associated or in close proximity to genes involved in stumpy formation or post-transcriptional regulators suggesting an interesting and yet unexplored functional link between them. If this holds true, it would also mean that tagging some specific proteins from the endogenous locus - a popular approach in this field - (where UTRs are disrupted) may have unforeseen functional consequences. The authors also identified 11 novel protein coding genes. Since both NGS and mass spectrometry rely heavily on databases, I find this manuscript a helpful contribution to the continuous curation of the *T brucei *genome. I also celebrate how detailed the Methods section is. I am supportive of publication, though make some suggestions for possible improvement of the manuscript.

**Major criticism:**

1. lncRNAs:
  The author found that several lncRNA are in close proximity (typically at the 5’-end) to genes associated with the transition between the BSF and PCF life stages or potential post-transcriptional regulators. Is this statically significant? How does it compare to different functional gene categories or among developmentally vs non-developmentally-regulated genes? The authors show the FC between different fractions but I would like to see how levels of the protein-coding gene and its proximal lncRNA vary in PF vs BF. For instance, what is going on with the lncRNA immediately upstream of the RBP10 gene in PF? Since it seems to be embedded into the 3’UTR of the upstream gene (Tb927.8.2770), is its level independently regulated or follow the Tb927.8.2770 pattern? Most of the lncRNAs are found in subpolysomal fractions. For the nuclear grumpy it is expected. However, RBP7A seems to be cytosolic (also RBP10 for which a subpolysomal lncRNA is apparently linked), so how does the author imagine the crosstalk between the coding genes and their proximal lncRNAs? 
2. In the Discussion, the author hypothesized that the excised lncRNA might be targeted for degradation, and this could be the reason why the lncRNAs are enriched in the sub-polysomal fractions. Fig 15 shows that the lncRNA present upstream of the RBP10 coding region is indeed detected in the ribosome profiling data in both PF and BF stages (although no peptides detected). In human cells, cytoplasmic lncRNAs may indeed be recruited to ribosomes for degradation (PMID: 27090285). ^1^ Can this also apply for *T. brucei *and what is observed are lncRNA that are for instance either nuclear (like grumpy) or on the way to ribosomes? The authors should discuss all the possible options and do comment about the presence of lncRNA-derived reads on ribosome profiling data. 
3. Novel proteins:
  Are the novel proteins developmentally regulated? Could the authors go back to the RITseq from Alsford et al. and check whether those novel genes are required for proliferation? 

 

**Minor issues:**

1. Is Vasquez *et al.*; not Vasquex *et al.*
2. The reference labelled as #7 may be the #12? 
3. In the Discussion, the authors claim that grumpy regulates the transformation from slender to stumpy forms; although the cited reference presents very interesting data, it is still preliminary. I would rephrase it in order to stress it down

Is the work clearly and accurately presented and does it cite the current literature?

Partly

If applicable, is the statistical analysis and its interpretation appropriate?

Yes

Are all the source data underlying the results available to ensure full reproducibility?

Yes

Is the study design appropriate and is the work technically sound?

Yes

Are the conclusions drawn adequately supported by the results?

Partly

Are sufficient details of methods and analysis provided to allow replication by others?

Yes

Reviewer Expertise:

Molecular Parasitology

I confirm that I have read this submission and believe that I have an appropriate level of expertise to confirm that it is of an acceptable scientific standard, however I have significant reservations, as outlined above.

## References

[1] : Cytoplasmic long noncoding RNAs are frequently bound to and degraded at ribosomes in human cells. *RNA* .22(6) : 10.1261/rna.053561.115 867-82 10.1261/rna.053561.115 27090285PMC4878613

# Response

**Published:** 2022-01-21

**Authors:** Michele Tinti

**Affiliations:** Dundee University, UK

We would like to thank the reviewer for their time giving feedback and providing constructive criticisms to the paper. Here we address the points raised in the reviewer comments. These are also reflected in the revised version of the manuscript.

  

1. The author found that several lncRNA are in close proximity (typically at the 5’-end) to genes associated with the transition between the BSF and PCF life stages or potential post-transcriptional regulators. Is this statically significant?

We acknowledge that we should determine the statistical significance (if any) of lncRNA proximity to genes known to be involved in post transcriptional regulation. To this end, we performed GO term enrichment analysis of the genes at the 5’ of the lncRNA (see new chapter: Expression Analysis of lncRNAs and surrounding genes, Figure 17 and associated text). [MF(1] [MT(2] 

  

1. How does it compare to different functional gene categories or among developmentally vs non-developmentally-regulated genes?

Guegan et al., 2020 reported that 19 *T. brucei* lncRNAs genes were located immediately upstream or downstream of 18 of the 43 SIF pathway genes. As discussed above, we have revised [MF(3] [MT(4] the paper adding a more robust statistical analysis and looking at GO enrichment analysis (see new chapter: Expression Analysis of lncRNAs and surrounding genes, Figure 17 and associated text). [MF(5] [MT(6] .

  

1. The authors show the FC between different fractions but I would like to see how levels of the protein-coding gene and its proximal lncRNA vary in PF vs BF. 

This is an interesting point. However, several lncRNA overlap with the mRNA transcripts. To properly answer this interesting point, we re-mapped our data considering only lncRNAs and the CDS of protein coding genes. We then added more robust statistical analysis of the lncRNAs and the genes at their 5’ or 3’ (see new chapter: Expression Analysis of lncRNAs and surrounding genes, Figure 17 and associated text).

  

1. so how does the author imagine the crosstalk between the coding genes and their proximal lncRNAs? 

We do not have a working hypothesis at the moment, and we make this clear in the revision.

  

1. Are the novel proteins developmentally regulated? Could the authors go back to the RITseq from Alsford et al. and check whether those novel genes are required for proliferation? 

With respect, we think that these points go beyond the scope of our analysis. We mostly just wanted to flag that a few more putative protein-coding genes exist to the trypanosome research community so that individuals can ask these types of questions .

**Minor issues:**

  

1. Is Vasquez et al.; not Vasquex et al.

We correct the misspelling.

  

1. The reference labelled as #7 may be the #12? 

We correct this miss annotation.

  

1. In the Discussion, the authors claim that grumpy regulates the transformation from slender to stumpy forms; although the cited reference presents very interesting data, it is still preliminary. I would rephrase it in order to stress it down.

We have changed the text to address this issue

# Peer review: Reviewer response for version 1

**DOI:** 10.21956/wellcomeopenres.18079.r42787

**Published:** 2021-03-15

**Authors:** Shulamit Michaeli

**Affiliations:** 1 The Mina and Everard Goodman Faculty of Life Sciences and Advanced Materials and Nanotechnology Institute, Bar-Ilan University, Ramat-Gan, Israel

The paper by Tinti *et al.* determined the RNA present in polysomal and sub-polysomal fractions by RNA-seq in the two life stages of the trypanosome parasites, PCF and BSF. The study compared their data to published ribosome-profilling and to the polysomal mRNA described by the Clayton group. This is an important study that detects interesting 5’ lncRNA. They propose an interesting hypothesis that part of long transcripts are excised out and moving part of mRNA to the sub-polysomal mRNA. They also detected snoRNA in the sub-polysomal fractions and their interpretation is that these are precursors en-route to be degraded. In addition, they describe new protein coding genes.

**Major criticism**

 The authors suggested based on the paper present in Archives that the 5’ lncRNA regulates the downstream gene (ref 31). However, even in the cited paper, there is no direct evidence that the lncRNA regulates the neighboring gene (RBP7A and B genes). There is no evidence that the ncRNA affects the expression of the neighboring genes also in this stud. In the cases presented in Fig. 14, 15, and 16 and especially in Fig. 14, the CDS and the upstream lncRNA are in my eyes individually trans-spliced and polyadenylated transcripts.

 So these could be independent entities. The lncRNA may even regulate other transcripts. To convince that there is a continuous transcript between the lncRNA and the downstream gene it is needed to amplify an RNA with primers coming from both genes that is trans-spliced and polyadenylated. Without this experiment, one can not say that the 5’ UTR lncRNA is processed from a longer transcript carrying the CDS.

 Regarding the snoRNAs, snoRNA are indeed processed from transcripts that are trans-spliced and polyadenylated but are processed by endonucleolytic cleavage. There is no presentation of the snoRNA transcript reads to see the sequence of the intergenic regions (between the snoRNAs) so is not clear to me what the authors refer to i.e. snoRNA precursors or just snoRNA associated with RNP complexes. The association of snoRNAs with complexes from 90 to 60S is expected because snoRNAs are involved in ribosome processing and modification and hence are found in large processing complexes that are smaller in size compared to polysomes and are special processing complexes. 

 The issues presented above need to be clarified before this paper can be published.

Is the work clearly and accurately presented and does it cite the current literature?

Yes

If applicable, is the statistical analysis and its interpretation appropriate?

Yes

Are all the source data underlying the results available to ensure full reproducibility?

Yes

Is the study design appropriate and is the work technically sound?

Partly

Are the conclusions drawn adequately supported by the results?

No

Are sufficient details of methods and analysis provided to allow replication by others?

Yes

Reviewer Expertise:

Molecular biology and RNA Biology of trypanosomes

I confirm that I have read this submission and believe that I have an appropriate level of expertise to confirm that it is of an acceptable scientific standard, however I have significant reservations, as outlined above.

# Response

**Published:** 2022-01-21

**Authors:** Michele Tinti

**Affiliations:** Dundee University, UK

We would like to thank the reviewer for their time giving feedback and providing constructive criticisms to the paper. Here we address the points raised in the reviewer comments. These are also reflected in the revised version of the manuscript.

  

1. We agree with the referee when she states that “So these (lncRNAs) could be independent entities. The lncRNA may even regulate other transcripts”. In our paper, tried to be cautious about over-stating this as a working hypothesis. However, we thought it worth flagging to the scientific community the proximity of lncRNAs to genes regulating the life stage transitions in T. brucei and thus speculated on their possible roles in regulating gene transcription. In the revised version, we have removed this hypothesis.
2. The reviewer points out that “it is not clear to me what the authors refer to snoRNA precursors or just snoRNA associated with RNP complexes”. We referred to gene annotated as snoRNA in TriTrypDB. It would be hard to discriminate wherever they come from, if precursor or snoRNA associated with RNP complexes.
